# Characterization of the naive murine antibody repertoire using unamplified high-throughput sequencing

**DOI:** 10.1371/journal.pone.0190982

**Published:** 2018-01-10

**Authors:** Trisha A. Rettig, Claire Ward, Bailey A. Bye, Michael J. Pecaut, Stephen K. Chapes

**Affiliations:** 1 Division of Biology, Kansas State University, Manhattan, Kansas, United States of America; 2 Division of Biomedical Engineering Sciences, Loma Linda University, Loma Linda, California, United States of America; Oklahoma State University, UNITED STATES

## Abstract

Antibody specificity and diversity are generated through the enzymatic splicing of genomic gene segments within each B cell. Antibodies are heterodimers of heavy- and light-chains encoded on separate loci. We studied the antibody repertoire from pooled, splenic tissue of unimmunized, adult female C57BL/6J mice, using high-throughput sequencing (HTS) without amplification of antibody transcripts. We recovered over 90,000 heavy-chain and over 135,000 light-chain immunoglobulin sequences. Individual V-, D-, and J-gene segment usage was uniform among the three mouse pools, particularly in highly abundant gene segments, with low frequency V-gene segments not being detected in all pools. Despite the similar usage of individual gene segments, the repertoire of individual B-cell CDR3 amino acid sequences in each mouse pool was highly varied, affirming the combinatorial diversity in the B-cell pool that has been previously demonstrated. There also was some skewing in the V-gene segments that were detected depending on chromosomal location. This study presents a unique, non-primer biased glimpse of the conventionally housed, unimmunized antibody repertoire of the C57BL6/J mouse.

## Introduction

B cells are an important part of the adaptive immune system, arising from hematopoietic stem cell precursors. These cells express surface immunoglobulin (Ig) receptors and secrete these same proteins as antibodies into the serum after differentiation into plasma cells [1, 2].

As B cells develop, they rearrange Variable- (V), Diversity- (D), and Joining- (J) gene segments, which combine with a constant region to form the antibody structure [3, 4]. Antibodies consist of heterodimers of heavy and light chains [4]. The heavy chain is formed from V-, D-, and J-gene segments combined with a constant region [5], while light chains lack a D-gene segment. [3, 6].

There are three complementarity determining regions (CDR). CDR1 and CDR2 are encoded in the V-gene segment. CDR3 consists of a combination of V-, (D-, heavy-chain), and J-gene segments [7]. Of the CDRs, CDR3 contributes the most to binding specificity. Antibodies are further characterized by the constant region, or isotype, which is influenced by the stage of B-cell development and antigen specificity [8].

The total collection of antibody specificities present within an individual is known as the antibody repertoire. Diversity of the antibody repertoire results from four main components: the initial germ line (inherited), diversity from recombination of that germline, the imprecisions during V(D)J recombination, and somatic mutations [9–11]. The antibody repertoire has been examined in many studies by high-throughput sequencing (HTS) and fully mapped in the zebrafish [12].

Repertoires can serve as a fingerprint or snapshot of the current immune-system status and these types of data have been used to explore the development of host defense to infectious disease [13–18], cancer [19–22], autoimmune disease [23, 24], and early disease detection [25]. With the development of HTS, we are now able to detect the differences between or among B-cell repertoires such as B2 (adaptive antibodies) and B1 (natural antibodies) B cells [11] or memory and naïve repertoires [26, 27]. HTS has accelerated the characterization of the widely differing human Ig haplotypes [28–32], and strain-specific gene segment usage in mice [33].

Our long-term goals are to investigate the repertoire of B cells in mice in space and how it changes in response to antigen challenge. More specifically, our lab is interested antibody repertoire dynamics within the context of spaceflight. Due to the cost of these experiments, creating datasets that can be mined by our lab or others is important. The antibody repertoire is traditionally assessed through the amplification of Ig sequences that have been isolated from sorted B cell populations [34]. While these practices increase the likelihood of recovering rare Ig sequences and allow for the dissection of the antibody repertoire by B-cell populations, cell sorting may not be possible within the design of certain experiments. During the development of methodology to assess Ig-gene usage by mice subjected to space flight we performed multiple HTS runs to validate sample preparation, bioinformatic methodology, and reproducibility [35]. We also wanted some background data about the Ig repertoire in the normal B6 mouse population. Knowing that there can be significant mouse-to-mouse variability in the Ig repertoire [36, 61] and to minimize the impact any one mouse might have in the validation data set, we chose to pool multiple mice specifically for these validation experiments [35]. We now present these data on the splenic repertoire of conventionally housed, unimmunized, unchallenged, adult C57BL/6J mice.

## Materials and methods

### RNA extraction and sequencing

Tissue extraction and sequencing were performed as described previously [35]. Briefly, spleens were collected from three independent pools of four, specific pathogen-free (based on the RADIL Advantage Basic profile), female, C57BL/6J mice nine-to-eleven weeks old. Animals were euthanized with isoflurane overdose followed by cervical dislocation. Briefly, mice were exposed to 400μL isoflurane in a gauze-pad enclosed in a histopathology cassette in a 450 mL chamber as was described by Huerkamp *et al* [37]. Animals used in pool one were raised in the Laboratory Animal Care Services (LACS) facility (breeder stock renewed less than 2 years previous) in the Division of Biology at Kansas State University. Mice from pools two and three were received directly from Jackson Laboratories and were house in the LACS facility. Mice were fed LabDiet 5001 and had access to water and food *ad libitum*. Mice were maintained on a 12/12 light/dark cycle. Mice for pools two and three were allowed to acclimate in the vivarium for 22–31 days prior to sacrifice. Animal procedures were approved by the Institutional Animal Care and Use Committee at Kansas State University. After euthanasia, spleen tissue was processed immediately for RNA extraction with Trizol LS according to the manufacturer's instructions. Pool one contained RNA from one-half of the spleen tissue while pools two and three contained RNA extracted from complete spleens. Equal concentrations of total splenic RNA (RIN>8) were pooled and mixed from each of the four mice, resulting in three final pools. At least one microgram of RNA from each pool was submitted for size selection (275–800 nucleotides) and sequencing on Illumina MiSeq at 2x300 nucleotides as described previously [35]. Sequencing was performed at the Kansas State University Integrated Genomics Facility using the standard Illuimna sequencing protocol, including oligo-dT-bead selection of mRNA and reverse transcription to cDNA. A reduced fragmentation time (one minute) was used to yield longer transcripts [35]. To avoid potential primer bias and maintain a dataset that could be further mined, we did not amplify Ig sequences. The authors note that a subset of mouse pool one data was used in a methods paper presented by our group. The context and focus of that previous manuscript does not overlap with the current work [35].

### Bioinformatics

Sequence selection, mapping, and final processing was performed as outlined previously [35]. Briefly, sequences were imported into CLC Genomics Workbench v9.5.1 (https://www.qiagenbioinformatics.com/) and cleaned to obtain high quality reads using a quality score of 97% of the sequencing containing a Phred score over 20. Both paired and merged (overlapping pairs) were mapped to V-gene segments and the loci using a match score of +1 and a mismatch score of -2 to identify potential antibody sequences. The sequences were collected and submitted to ImMunoGenTics’s (IMGT) High-V Quest for identification [38]. Due to the chances of collecting the same read multiple times through the mapping and identification process, only one sequence per sequence ID was analyzed (procedure outlined in Rettig *et* al [35]). Productive and unknown functionality sequences were identified via IMGT and used for subsequent analyses. Productive antibody sequences were defined as in frame and did not contain a stop codon. However, binding abilities were not assessed. Unknown sequences did not contain enough sequencing information to determine functionality. Gene segments were identified using IMGT’s nomenclature and using IMGT’s list of gene segments, including functional and open reading frame-defined gene segments. We implemented two procedural changes to further define the repertoire that are different from the methodology presented in Rettig *et al*. [35]. In this current manuscript, when calculating the percent abundance in the repertoire, we also include V-gene segments where one or two possible V-gene segments were detected. When one single V-gene segment was detected in a sequence, it was assigned a value of one. When two potential V-gene segments were detected each gene segment was assigned a value of 0.5. The totals were then tabulated as described in Rettig *et al* [35]. Additionally, CDR3 sequences that did not fit the C-xx-W motif for IgH (heavy-chain) were reclassified as unknown functionality, unless a class-switched isotype (IgA, IgD, IgE) was detected. CDR3 sequences for Igκ (kappa-chain) that did not fit the C-xx-F motif were classified as unknown functionality. Sequencing reads containing hyperlengthy (greater than 2x the average, or 18 amino acids (AAs)) κ-CDR3 that fit the C-xx-F motif were also removed from analysis as we believed they were falsely identified through bioinformatic or sequencing errors.

Initial nucleotide alignments were created with MAFFT [39] using portions of the germline and CDR3 nucleotide sequences provided by IMGT. Sequences were sorted by identity, compared to the germline and the sequence order was then adjusted to group similarly-aligned sequences. Nucleotide sequences of identical length were then isolated from the full alignment and aligned with each other while retaining all previously-inserted gaps.

### V(D)J pairing frequency

Pairing frequency was assessed in productive sequencing reads from both IgH and Igκ datasets. All pairing of V-gene segments was only assessed from productive IgH and Igκ sequencing reads, referred to as VH and Vκ, respectively. Sequences identifying more than one possible V-gene segment were excluded from this analysis. For both IgH and Igκ, J- and D-gene segments designated as undetermined (U) were either not reported by IMGT, contained less than six nucleotides, or multiple gene segments were assigned to a single sequencing read. Total counts from VJ pairings for heavy- and light-chains were tabulated and Circos graphs were generated using Circos Online [40].

### Statistical analysis and graphics

Linear Regressions were performed by comparing the percent of repertoire of V-gene segment or V(D)J combinations from pools 1 vs 2, 2 vs 3, and 1 vs 3 using the linear regression analysis tool in GraphPad (Version 6.0). Chi-square analysis of V-gene segment usage was performed on raw sequencing read counts using R version 3.4.2 (https://www.r-project.org/). All productive VH- and Vκ-gene segments and open reading frames listed on IMGT for the B6 mouse were analyzed and gene segments not found in our datasets were assigned a read count of zero. The IMGT productive list includes all gene segments detected in the NCBI annotation of the mouse genome. The IMGT chromosomal locations were also used for any chromosomal analysis, though not all gene segments have a defined chromosomal location. Gene segments that were not defined by location in IMGT were excluded from any chromosomal analysis. These analyses were performed on each mouse pool separately by comparing the observed raw-read count values of V-gene segments to an expected theoretical number of reads which was based on the null hypothesis that all V-gene segments will have the same number of raw reads. This value was determined by dividing the total number of antibody reads observed in a mouse pool by the number of possible gene segments. The analysis of gene-segment usage by chromosomal location was performed by dividing gene segments into four quadrants based on nucleotide position. A quadrant was defined as one-fourth of the entire locus, as determined by number of nucleotides in the locus. Gene segment location was defined as the first nucleotide of the gene segment. A Chi-square analysis was performed on each mouse pool by summing the raw-read counts of all gene segments containing a 5’ nucleotide position within each quadrant and comparing the observed total reads within a quadrant to an expected theoretical number of reads for each quadrant which was based on the null hypothesis that the number of raw reads is not statistically different between defined quadrants.

Percent of repertoire values were determined by dividing sequencing reads corresponding to each gene segment, constant region, or CDR3 length by the total number of gene segments, constant regions, or CDR identified in each mouse pool for normalized comparison between pools. Percent of repertoire for these variables were displayed as bar graphs, generated in GraphPad v6.0, or as heat maps, generated in Microsoft Excel. In addition to visualization of gene segment combinations through Circos graphs, the percent of repertoire for gene segment pairings was also visualized using the bubble chart tool in Microsoft Excel.

## Results

### VH- and Vκ-gene segment usage

**Fig 1:**
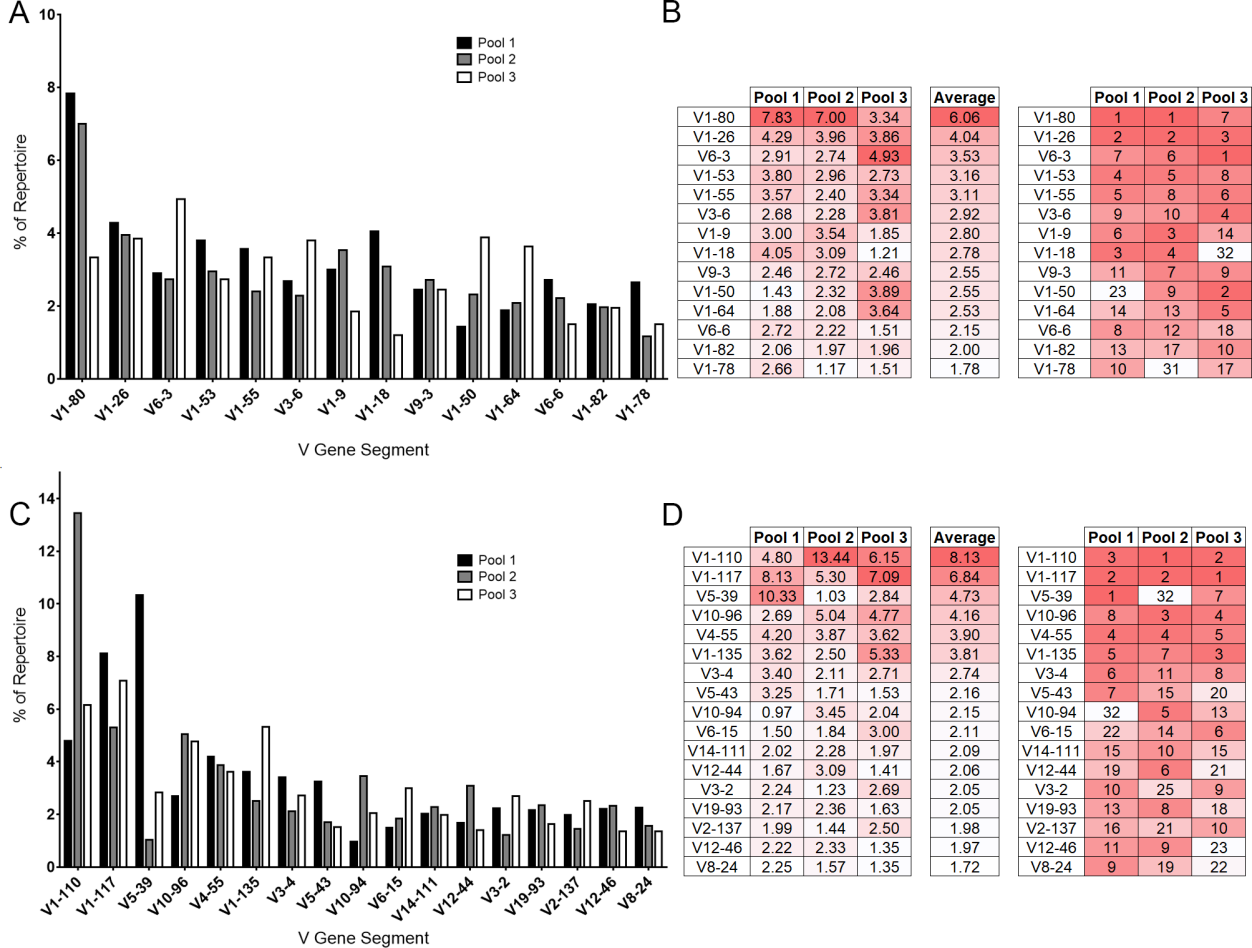
V-gene segment usage among unimmunized mouse pools. Sequencing reads mapped to each individual gene segment were divided by the total sequencing reads of all identified gene segments from each mouse pool for a normalized comparison between pools. (A) The VH representing the ten most abundant gene segments from each mouse pool are displayed. (B) The rankings of each gene segment contained within the top 10 most abundant VH from at least one of the mouse pools are compared. The most abundant gene segment is ranked as 1. Dark red indicates higher rank moving to white, of lower rank. Similarly, the top 10 abundant Vκ are displayed (C-D).

We obtained between 8,714 and 11,200 IgH individual productive reads, and between 14,271 and 28,756 individual IgH reads of unknown functionality as identified by IMGT HighV-Quest (Table 1). Between 11,968 and 18,643 individual Igκ productive, and 12,602 and 39,410 individual Igκ reads of unknown functionality were identified by IMGT HighV-Quest (Table 1). Overall, we identified 132 VH- and 109 Vκ-gene segments within the repertoires of our mouse pools (S1 Fig). As a general trend, the three pools resulted in similar frequencies and similar ranks for V-gene usage among groups. The ten most common V-gene segments from each pool were compiled, resulting in a total use of 14 VH-gene segments and 17 Vκ-gene segments (Fig 1).

**Table 1:** Sequencing and mapping statistics from mouse pools 1, 2, and 3.

	Pool 1	Pool 2	Pool 3
Total Reads	25.1 M^a^	31.4 M	32.7 M
Post Cleaning	12.0 M	30.9 M	32.0 M
Productive IgH	8,714	11,200	10,224
Unknown IgH	14,271	27,896	18,756
Productive Igκ	11,968	18,643	16,293
Unknown Igκ	12,602	39,410	36,624

^a^M: million

The most common VH-gene segment in pools one and two was V1-80 (Figs 1A and S1A). V1-80 was the seventh most common gene segment used in pool three. V6-3 was the most common VH-gene segment in pool three, but ranked seventh and sixth in pools one and two, respectively (Fig 1B). V1-26 was the next most common VH-gene segment, ranking second in pools one and two, and third in pool three. Among the top ten most common VH-gene segments, most gene segments ranked between first and 17th within their pools, however, three outliers were found within these groups. V1-50 ranked 23rd in pool one, but it was ninth and second in pools two and three, respectively. V1-78 was ranked 31st in pool two, but ranked tenth and 17th in pools one and three, respectively. V1-18 was ranked 32nd in pool three but third and fourth in pools one and two, respectively. VH-gene usage between pools was well correlated (1 vs 2 R^2^ = 0.8427, 2 vs 3 R^2^ = 0.7054, 1 vs 3 R^2^ = 0.5842, all p = <0.0001).

Seventeen Vκ-gene segments were among the top 10 most abundant Vκ of the repertoire in at least one of the three mouse pools, with five Vκ-gene segments appearing in the top ten of all three mouse pools (Figs 1 and S1B). Pool one appeared enriched for V5-39, comprising 10.33% of the repertoire as compared to 1.03% in pool two and 2.84% in pool three (Fig 1C). Excluding this difference, V1-110, V1-117, V10-96, and V4-55 were the four most abundant gene segments in all three mouse pools. The lowest ranking of the most abundant Vκ in any of the mouse pools were V5-39, ranking 32^nd^ in pool two, while ranking first and seventh in pools one and three, respectively and V10-94, ranking 32^nd^ in pool one, but fifth in pool two and 13^th^ in pool three. (Fig 1D). All top Vκ that showed any variation in abundance among pools were still within the top 32 Vκ-gene segments. Vκ usage between pools was correlated, although not as highly as VH (1 vs 2 R^2^ = 0.3684, 2 vs 3 R^2^ = 0.6700, 1 vs 3 R^2^ = 0.6414, all p = <0.0001).

**Fig 2:**
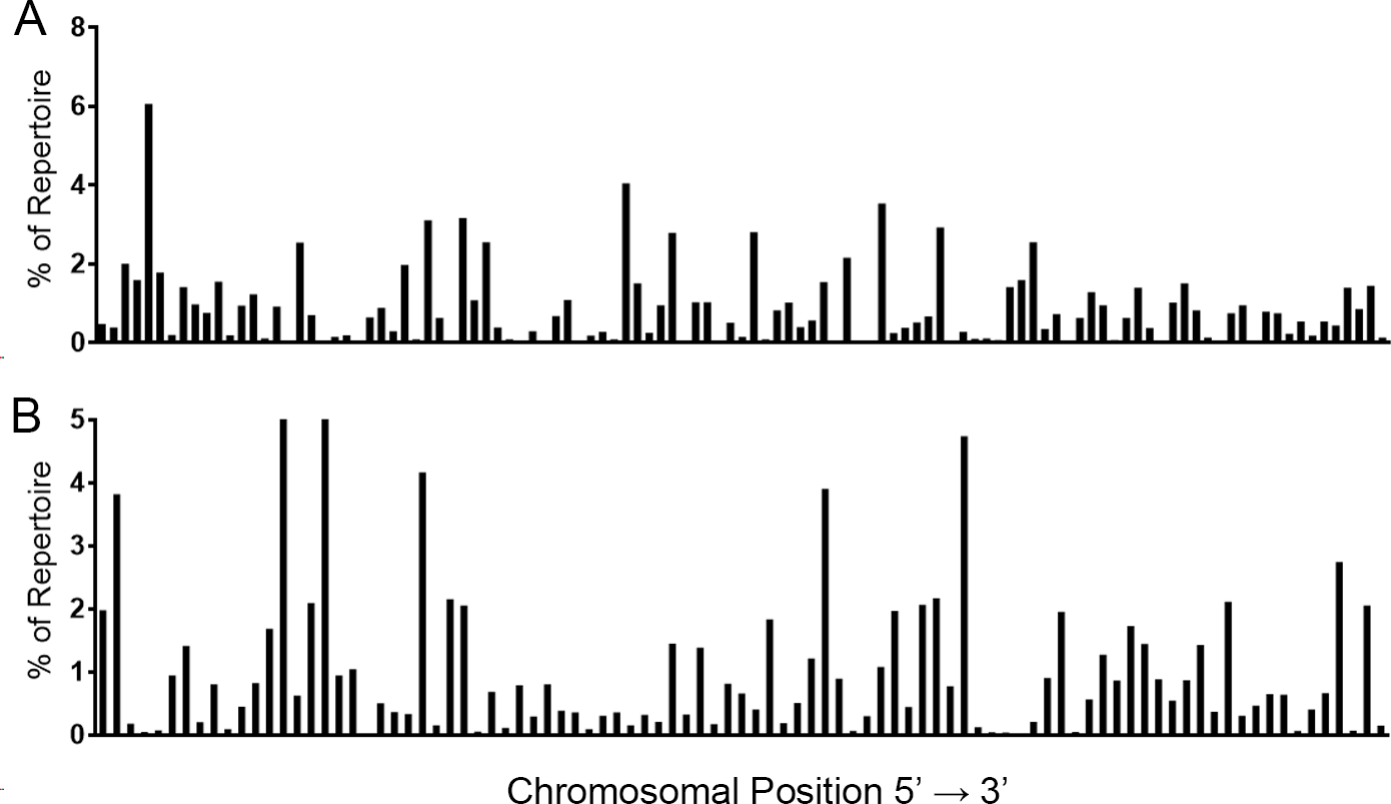
**V-gene segment usage among unimmunized mouse pools for IgH (A) and Igκ (B) by chromosomal location.** Gene segments are shown in order of chromosomal position (5’ to 3’). The average value from three mouse pools for each CDR3 length is shown. Distribution was assessed via Chi-square analysis in R (version 3.4.2) (all pools, p<0.0001).

With 113 productive VH and 93 productive Vκ described in IMGT by chromosomal location, each gene segment would be expected to appear as part of the repertoire roughly 0.84% and 1.06% of the time for VH and Vκ, respectively, if gene segment usage was random (Fig 2). When we assessed usage frequency, there was a non-random distribution of both VH- (Fig 2A) and Vκ- (Fig 2B) gene segments (Chi-square analyses; all pools p<0.0001).

We also assessed gene segment usage by chromosomal location in the IgH and Igκ loci as gene-segment spacing was not evenly distributed along the chromosome. Based on 5’ nucleotide position, Q1-4 contained 22, 23, 33, and 33 VH-gene segments or 18, 21, 24, and 30 Vκ-gene segments, respectively. Since V-gene segment usage appears skewed, we tested whether the total expression of V-gene segments within a quadrant defined by nucleotide position was randomly distributed. We found that the expression within each quadrant was not randomly distributed, for both IgH and Igκ, suggesting that V-gene expression may be influenced by chromosomal location (all pools, p<0.0001).

### DH-, JH-gene segment and IgH constant region usage

**Fig 3:**
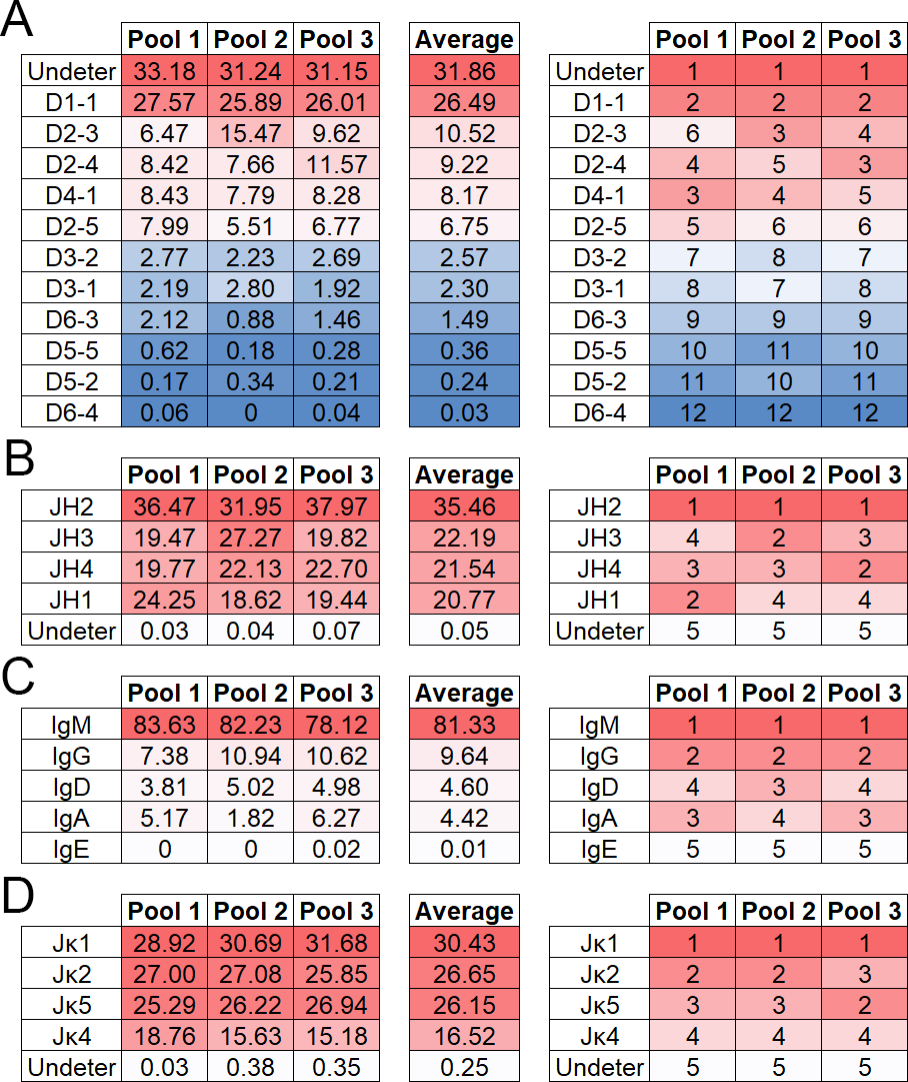
**Percent abundance of IgH D- (A) and J- (B) gene segments, IgH constant regions (C) and Igκ J-gene segments (D).** Sequencing reads corresponding to each gene segment or constant region were divided by the total number of gene segments or constant regions identified in each mouse pool for normalized comparison between pools (left side). The most abundant gene segment is ranked as one (right side). Dark red indicates higher rank moving to blue (A) or white (B-D), of lower rank. Sequencing reads designated undetermined (undeter) where portions of a D- or J-gene segment were identified but unable to be assigned to a specific C57BL/6J D- or J-gene segment.

We identified ten different D-gene segments used in our repertoires (Fig 3A). We also added one additional category for our analyses, termed “undetermined”. This label was applied to D-gene segments that were assigned by IMGT to non-C57BL/6J genes and antibody sequences containing a V- and J-gene segment, but not containing an identifiable D-gene segment. Due to the very short length of D-gene segments combined with alterations during recombination, D-gene segments were bioinformatically difficult to identify.

For all three groups, the D1-1 gene segment was the most common segment identified comprising 26–28% of the repertoire. Undetermined D-gene segments, however, made up a large part of the D-gene segment repertoire, comprising 31–33% of the repertoire. D2-3, D2-4, D4-1, and D2-5 were found in similar frequencies ranging from six to fifteen percent of the data set. D3-2, D3-1, D6-3, D5-5, D5-2, and D6-4 were found at low levels in all data sets; comprising under three percent of the total repertoire.

Four JH-gene segments were identified, with JH2 being the most common among all three groups (Fig 3B). The remaining J-gene segments, JH4, JH3, and JH1, were found at similar levels among groups totaling between 19% and 27% of the repertoire.

IgM was overwhelmingly the most commonly identified constant region making up between 78% and 84% of the total repertoire (Fig 3C). IgG was the next most common between seven and eleven percent of the total repertoire. IgA and IgD were rare totaling between two and six percent of the repertoire. IgE was only detected in pool three at less than one percent. It was not detected in pools one and two.

### Jκ-gene segment usage

A total of four Jκ-gene segments were identified, with similar distribution of Jκ-gene segments between mouse pools (Fig 3D). Due to the even distribution of the three most abundant Jκ, the ranking of each gene segment varied slightly among the three mouse pools. Within each mouse pool, there was a small portion of Jκ that contained too few nucleotides to be assigned to a specific gene segment (0.03–0.38%).

### IgH- and Igκ- gene segment combinations

**Fig 4:**
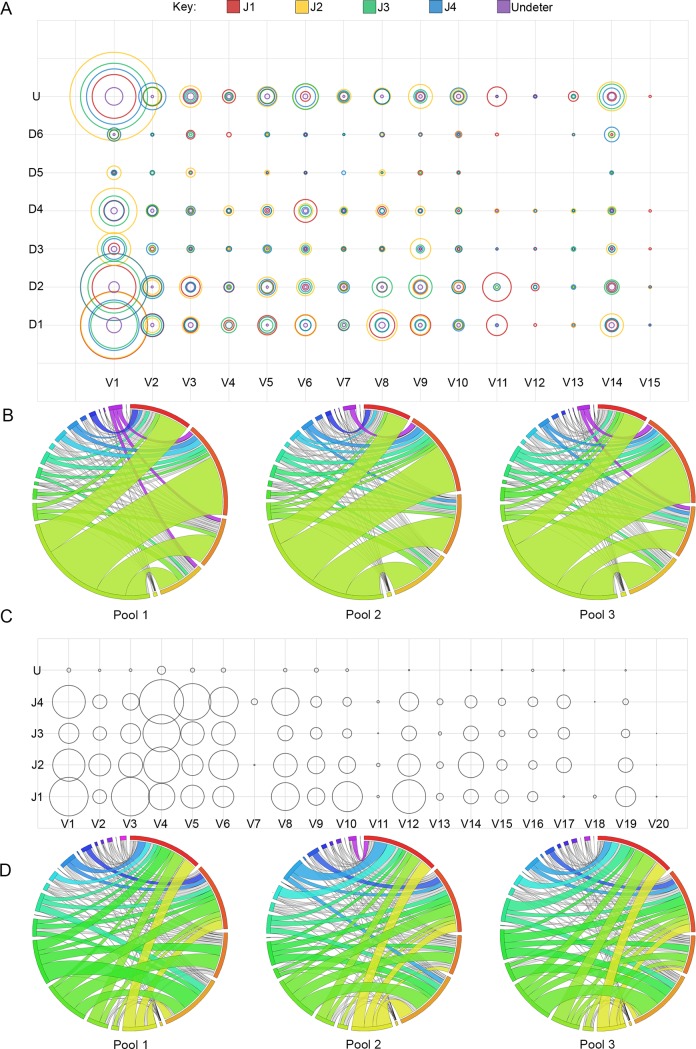
**Combinations of V-gene families with DJ-gene segments for IgH (A) and J-gene segments for Igκ (C).** Increasing pairing frequency of V(D)J is represented by larger circles. Sequencing reads in which more than one C57BL/6 J-gene segment was attributed or too few nucleotides were present in the J-gene segment for designation by IMGT have been classified as undetermined (U). Pairing frequency is also represented by Circos graphs for IgH (B) and Igκ (D). Circos Plot Labels (starting at 12:00 position and the largest arc and continuing clockwise with occasional color references) B–J1 (red), J2, J3, J4, U (yellow), V1, V2, V3, V4, V5, V6, V7 (Teal), V8, V9, V10, V11, V12, V13, V14 (purple), V15 D–J1 (red), J2, J4, J5, U, V1 (yellow), V2, V3, V4, V5, V6, V7 (black sliver), V8, V9, V10, V11, V12, V13, V14, V15 (royal blue), V16, V17, V18, V19, V20 (black sliver, if present).

VH, DH, and JH family combination frequency was examined. Some preferential bias for specific gene segments seemed to exist (Fig 4A). For example, the JH4/DH2 combination appeared at a high frequency with VH1 (4.5% of repertoire), but not with any other VH gene family to the same degree. IgH gene segment recombination frequency correlated with gene segment abundance. VH1, which contains over half of all possible V-gene segments, also was the most commonly used VH family, which is seen as the dominant band in the Circos plot (Fig 4B).

The pairing of Vκ families to individual Jκ was also assessed (Fig 4C and 4D). Overall, the pairing of Vκ families with Jκ appeared random, however, certain Vκ families preferentially paired with specific Jκ-gene segments. For example, V4 paired less efficiently with J1, while V3 paired more efficiently with J1 (Fig 4C). Unlike VH, no single Vκ family was exceedingly dominant. Although V4 was the most represented gene family, its expression level was close to that of the second next most prominent gene families, which varied by mouse pool as shown in the Circos plot (Fig 4D).

The percent of repertoire that each VJ-gene segment combination comprised within each mouse pool was compared by linear regression. Mouse pools showed modest correlation levels of VJ-gene segment recombination frequency in IgH (1 vs 2 R^2^ = 0.6055, 2 vs 3 R^2^ = 0.4419, 1 vs 3 R^2^ = 0.4399, all p = <0.0001) and Igκ (1 vs 2 R^2^ = 0.2340, 2 vs 3 R^2^ = 0.3598, 1 vs 3 R^2^ = 0.4607, all p = <0.0001), with some enrichment for certain combinations within each mouse pool. V-(D)-J combinations were within bubble charts generate a visual comparison of pairing (Fig 4A–4C).

### IgH and Igκ CDR3

**Fig 5:**
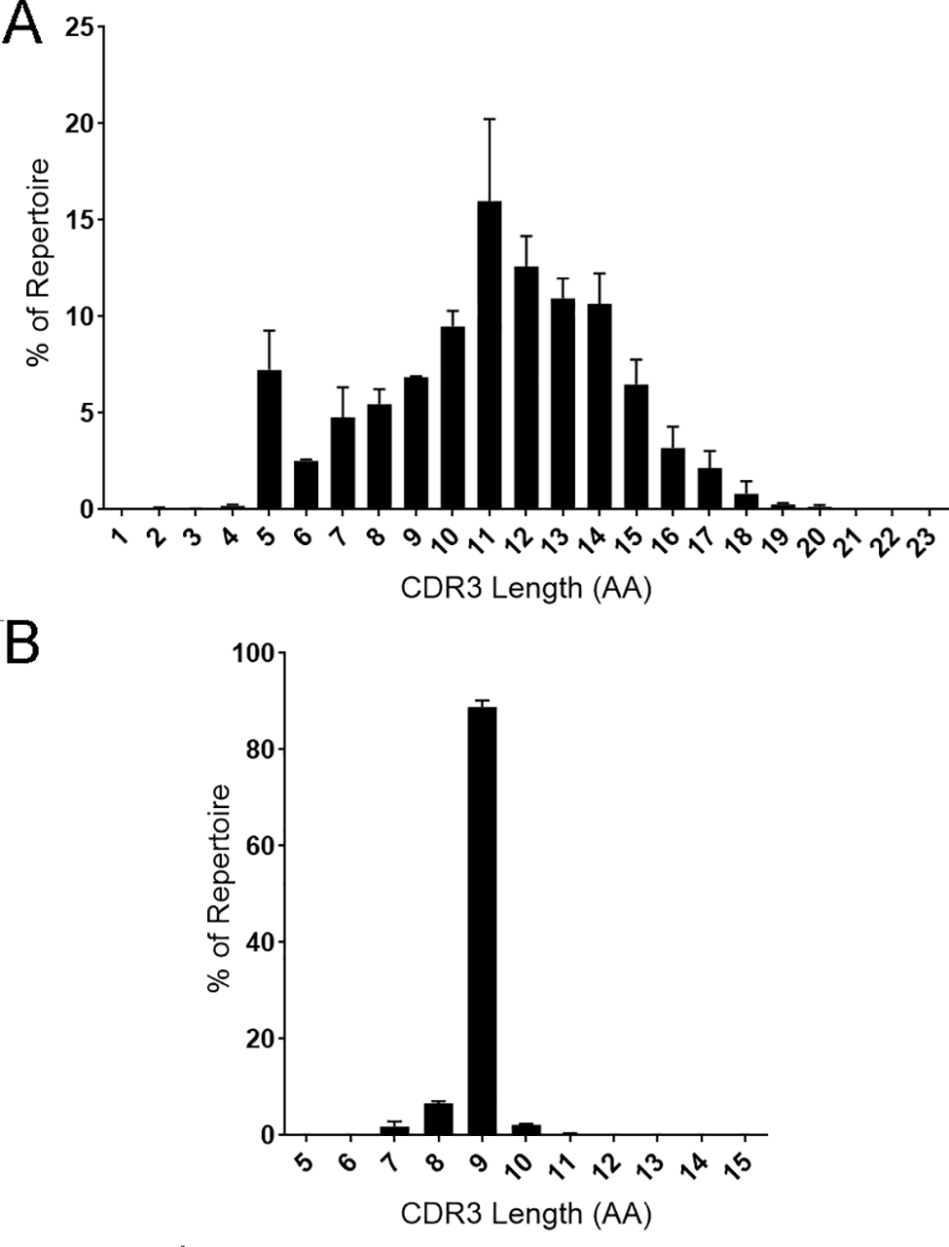
**CDR3 length for IgH (A) and Igκ (B).** The average percent of repertoire of each CDR3 amino acid length from three mouse pools is displayed.

The average IgH CDR3 (H-CRD3) length of all three data sets was 11 amino acid (AAs) long (Fig 5A). The lengths of the H-CDR3s followed a normal distribution except all three groups were enriched for five AA H-CDR3s. H-CDR3 AA length ranged from one to twenty-three amino acids in length with 11 AAs being the average for all three pools. Igκ chain CDR3 length was conserved at nine AAs, comprising 87–90% of the repertoire (Fig 5B). While nine AAs was the most frequent κ-CDR3 length, one κ-CDR3 with a length of seven AAs was observed in the top CDR3 sequences. The distribution of CDR3 lengths was even among pools for both IgH and Igκ (S2 Fig). Four κ-CDR3 sequences that fit the conserved kappa chain C-xx-F motif identified within the mouse pools (data not shown), while no such hyperlengthy H-CDR3 sequences fitting the C-xx-W motif were identified.

**Fig 6:**
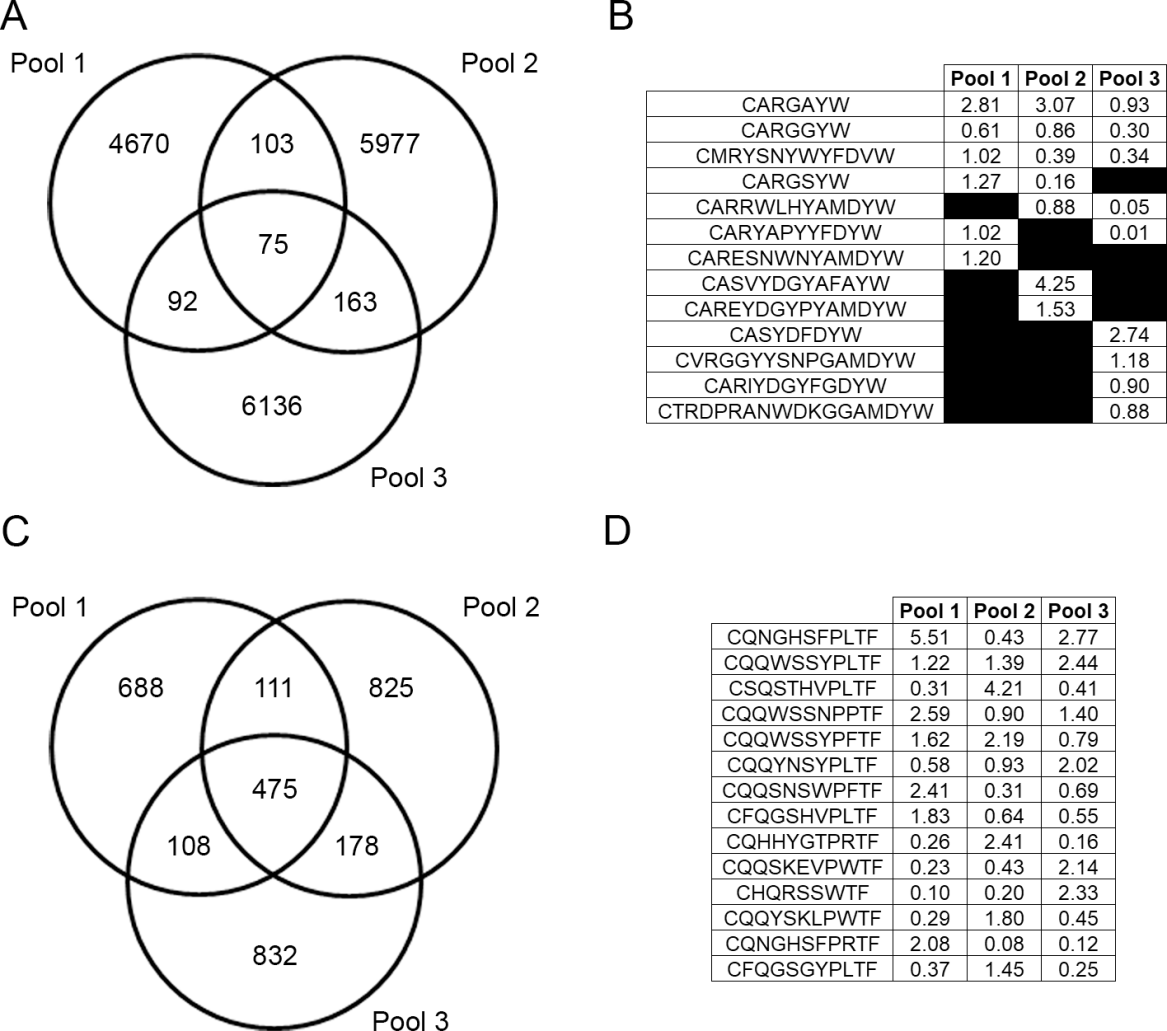
Top CDR3 AA sequences and overlap of unique CDR3 sequences within mouse pools. A Venn diagram displays the overlap of the number of unique CDR3 amino acid sequences among mouse pools for IgH (A) and Igκ (C). The percent of repertoire for the top five CDR3 amino acid from each mouse pool are shown for IgH (B) and Igκ (D).

A total of 17,216 unique H-CDR3 AA sequences were identified among all three data pools. Among those identified, the majority (16,783) were identified in only one pool (Fig 6A). Of the remaining CDR3s, 358 were identified in only two pools and 75 were identified in all three pools.

Interestingly, many of these H-CDR3s, though found in all three pools, were not necessarily common H-CDR3s. Only one H-CDR3, CARGAYW, was found among the top ten most common H-CDR3s of each pool. One additional H-CDR3, CARDYYGSSWYFDVW, was found in the top five of pools two and three. Of the 75 total H-CDR3s that appeared in all three pools, frequencies varied drastically, from being the most common to only being detected once (Fig 6A). Of the top five most common H-CDR3s in each data set, only three, CARGAYW, CARGGYW, and CMRYSNYWYFDVW occurred in all three data sets (Figs 6B and S3A). CARGSYW occurred in pools one and two, CARRWLHYAMDYW in pools two and three, and CARYAPYYFDYW in pools one and three. The remaining most common H-CDR3s occurred in only one pool. A heatmap of all 75 shared H-CDR3 is shown in S3A Fig.

There were 3,217 total unique κ-CDR3 amino acid sequences identified among all three mouse pools (Fig 6C). While there were 2,345 individual κ-CDR3 amino acid sequences that were unique to the individual mouse pools, there were also 475 κ-CDR3 shared among all three pools. None of these 475 shared κ-CDR3 were found within the top five κ-CDR3s of all three mouse pools (Fig 6D). There were 14 total κ-CDR3 sequences that were found within the top five κ-CDR3 for each mouse pool (Figs 6D and S3B). There were no CDR3 sequences that were found within the top five κ-CDR3 in all three mouse pools.

### Comparison of alignments of CDR3s

**Fig 7:**
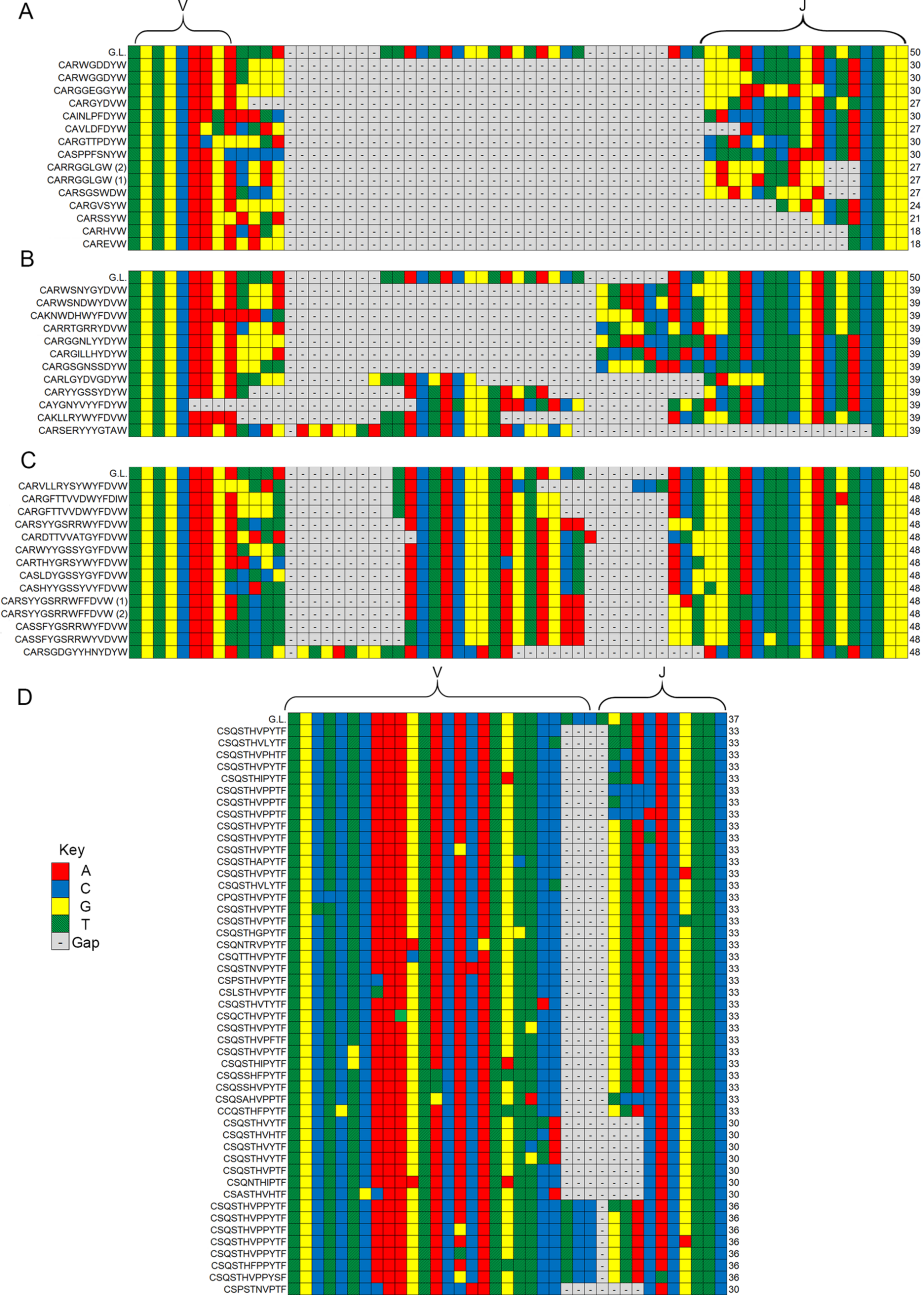
**Comparison of CDR3 alignments in gene segment combinations (IGHV1-26, IGHD1-1, IGHJ1) coding for a predominantly short (A), median length (B), and long (C) H-CDR3 region and κ-CDR3 (IGκV1-110, IGκJ-2) (D).** The germline nucleotide (G.L.) sequence is identified at the top of each alignment. Each nucleotide sequence is labeled with its corresponding amino acid sequence. Nucleotide sequences coding for identical amino acid sequences are labeled with numbers (1, 2, 3, etc.) corresponding with the alignment order. The V and J-gene segments for each alignment are labeled, however due to the variability in the D-gene segment it is not bracketed, but is identifiable by the germline sequence provided.

To assess the heterogeneity in B-cell idiotypes created by the differential splicing of Ig genes, we compared B cells that used the same V-, D- and J-genes. Two gene combinations containing complete V-, D-, and J-segments common to top 35 gene combinations found in all three mouse pools were selected for this analysis (Fig 7A–7C).

One heavy-chain VDJ-gene combination displaying a CDR3 region of variable length was selected from the 15 most common gene combinations among the three mouse pools and aligned to its germline sequence. From the full alignment, one short (four to eight AAs, Fig 7A), one medium (11 AAs, Fig 7B), and one long (14 AAs, Fig 7C) selection of nucleotide sequences were isolated and compared. Although the three groups were encoded by the same V-, D-, and J-gene segments, gene segment representation across each sample was variable. Most variability occurred in or around the D-gene segment, which could be due to splicing, N- and P-nucleotide additions, and deletions during somatic recombination. D-gene usage also appeared to be a factor determining CDR3 length. This was evidenced by increasing D-gene representation across CDR3 selections of decreasing length compared to the relative conservation of the V- and J-gene segments, though J-gene conservation seemed to decrease among extremely short CDR3s. Overall, the V-gene segment appeared to remain the most uniform.

While Igκ contains mostly CDR3 sequences that are nine amino acids in length, many highly abundant VJ-gene segment combinations (such as V110 and J2) contained CDR3s of multiple lengths. Unlike IgH, the alignments of VJ pairings were relatively uniform among Igκ as compared to the germline sequence in CDR3 sequences that were eight, nine and ten amino acids in length (Fig 7D).

## Discussion

To our knowledge, these data are the first unamplified sampling of the normal mouse antibody repertoire that has been described. Others have looked at Ig-gene segment usage with other strategies [26, 36, 41–43] but we wanted to determine if a straight-forward RNA-Seq approach would provide us with a reasonable assessment of B cell Ig-segment use without the limitations that amplification methods introduce (Rettig *et al*., In Revision).

To minimize potential single animal aberrations and repertoire skewing, we pooled splenic tissue of four unimmunized mice in three biological replicates. This approach was successful since we saw less variation with pooled samples compared to data sets that are made up of single mice [61]. Grieff *et al*. demonstrated that CDR3 and VDJ composition in pooled mouse samples were less polarized than that of an individually sequenced mouse subjected to antigen challenge [44]. Therefore, our data are consistent with that study. We also did the technical replication of material in pool one and found that there was good reproducibility (R^2^ = 0.7562) [35]. Therefore, the data in the individual normal mouse pools and a compiled summary of those data are a strong reflection of the normal mouse repertoire.

The most common VH-gene segment was V1-80, which was the most common in pools one and two. V6-3 was the most common in pool three. In selecting the ten most common VH-gene segments from each pool, we identified 14 different VH-gene segments, with heavy overlap among pools. All VH-gene segments isolated comprised between 1% to 8% of the repertoire. This VH V-gene variation is consistent with previous observations [25] but the pools ameliorated the extreme variations that was reported by that group. We also saw that Vκ-gene segment usage was comparable among mouse pools, with 17 gene segments comprising between 1% and 13% of the repertoire. Although we have found that gene segment use among all three pooled sample groups was similar, there were some differences. V5-39 was observed at a high frequency in pool one (10%) as compared to pools two (1%) and three (3%). This skewing could be the result of a mouse within pool one responding to a specific antigen that other mice did not respond to or more likely, represents the natural variability of mice [36]. Nevertheless, even pooling samples maintains the randomness of antibody gene selection and rearrangement and a pool of four individuals still has relative uniqueness.

Some have suggested that V-gene segment usage may be skewed [45]. Chi-square analyses of VH- and Vκ-gene segment use in our data set would support this contention since several VH and Vκ-gene segments were used more frequently than expected. Even though we have analyzed three independent biological samples made of pools of four mice, we recognize that an even larger data set will be needed to conclusively settle this discussion. Studies on humans have revealed similar V(D)J usage in spite of them being outbred populations [27] which also supports that there is some inherent selection in V-gene selection regardless of genetics. Additional studies looking at epigenetic changes or other transcriptional regulatory elements such as the characterization performed by Choi et al. [46] might also help understand mechanisms of V-gene segment selection.

For the D-gene segment, three usage levels were detected. D1-1 was the most used gene segment in all three pools; comprising around 26% of the total repertoire. Over 36% of D-gene segments could not be identified, likely due to the short length of the D-gene segment. Nevertheless, we do see different populations of antibodies even when they do share similar D-genes segments. Some have large D-gene segments where others have little recognizable sequence.

JH-gene segment usage was relatively uniform. J2 was the most common among all three pools, with over 32% use in the repertoire. J1, J3, and J4 were evenly represented among all three pools totaling between 19–27% of the repertoire. Gene segments with less than six nucleotides were unable to be identified and occur at less than 0.1% of the heavy-chain repertoire. Jκ-gene segment usage is somewhat evenly distributed among J1, J2, and J5 comprising between 25–32% of the repertoire, in agreement with the findings of Aoki-ota *et al*.[45], Lu *et al*. found a slightly different Jκ expression profile, possibly reflecting strain specific usage of Jκ [26]. As paralleled in the heavy-chain data, gene segments with less than six nucleotides were rare; occurring in less than 0.4% of the total repertoire.

Constant region usage in the heavy-chain was heavily dominated by IgM, which reflects the “naïve” status of our mice. Although IgM comprised over 78% of the total identified constant regions we did see the expression of IgG, IgA and IgD. IgE was rare, being detected in only pool three. However, when compared to serum data, even in naïve mice, there was a high level of circulating IgGs, which was not reflected in spleen tissue sequencing, which instead shows very high levels of IgM [47]. This could be due to a large B-cell population in the spleen that is not secreting antibody at high levels into the bloodstream [36].

We looked at the common H-CDR3 sequences among the three mouse pools. There was little overlap; with only 75 H-CDR3s detected in all three pools and between 92 and 163 common H-CDR3s when we just looked at two pools. We detected between 4.6k to 6.2k unique sequences found only in each respective pool. While we sampled a small fraction of CDR3s present in the total antibody repertoire, Lu *et al*. and Greiff *et al*. used primer amplification to enrich for IgH transcripts and still found high CDR3 variability among individuals [26, 36].Similarly, in a study comparing monozygotic twins, Glanville *et al*., also demonstrated that CDR3 profiles between the individuals were quite diverse despite similar gene family usage between the twins [48]. Therefore, the pooling methodology that we employed did not significantly diminish the detection of the unique CDR3 repertoires that individuals have.

When we examined the κ-CDR3 usage among the three biological samples, there was a higher proportion of common κ-CDR3s. Unique κ-CDR3s within each pool ranged from 688 to 832 CDR3 identified within all three pools and between 108 and 178 CDR3 identified in only two pools. One explanation for light-chain CDR3 length homogeneity may be selection due to light-chain editing that occurs during B-cell maturation and the need to be able to interchange the light chains.

The small numbers of overlapping CDR3 sequences among our three pooled samples suggests that significant variation in the idiotypes could develop, even within an inbred population of mice and reinforces the idea of unique generation of B-cell diversity in inbred and outbred populations [27, 48]. Moreover, we were curious if the size of the total pool of B cells could be estimated from our data. Using a model of capture-recapture methodology [49], the Chapman estimator, [50] and the number of common heavy-chain CDR3 sequences seen in each of our samplings, we estimated our B-cell pool to range from 1.5–14 x 10^6^ cells. If we assume that there are 5 x 10^6^ B cells in a nine to eleven-week-old female C57BL/6J mouse spleen, and our mouse pools were made up of four spleens, this estimate of the possible splenic B-cell pool is reasonably accurate, especially if we take into account some of the CDR3 sequences were detected multiple times (multiple B cells with the same IgH).

While CDR3 is commonly used to describe the antibody repertoire, many studies have reported the combinations of the V(D)J [20, 44, 51–53]. Compiling CDR3 nucleotide alignments allowed us to visualize the significance of individual gene segment involvement with the CDR3 in the context of specific V(D)J combinations. Sequencing outside of CDR3 also can reveal biologically relevant information about antigen binding and allows for further characterization of B-cell ontogeny. Our approach allowed us to identify V(D)J-gene segments in addition to the constant region and provided insight into the pairing of V-gene segments with (D)J-gene segments.

The information about the unchallenged Ig-gene repertoire also has other uses. It provides a comparative foundation when looking at host response to antigen [54] and has been used to isolated therapeutic antibodies. Antibodies for influenza in a mouse model and were a valuable tool in the detection of antigen specific responses [16, 34].

While a lack of amplification may extricate primer bias, we knew that it would come at the cost of potentially excluding rare B-cell clones. In humans, for example, a single clone may only comprise 0.1% to 0.3% of the repertoire [55]. We have explored the differences between samples that have and have not been amplified and found a moderate correlation (R^2^ = 0.5815, 0.5855, p<0.0001, Rettig *et al*., In Revision). While some of the differences arose from expected depth-of-sequencing issues, we unexpectedly found that discrepancies also resulted from gene segments being detected in the unamplified data set not detected in the amplified data sets (Rettig *et al*., In Revision). It is also important to note that the number of immunoglobulin reads detected in our RNASeq library equaled or exceeded those in other HTS studies [55]. Therefore, we are aware of the tradeoffs and benefits of the HTS strategy we have used.

Another issue which may affect the data presented stems from the use of whole spleen tissue rather than isolated B-cell populations [34]. Although our approach was necessary to accommodate requirements of a separate investigation [35], we are aware that the inclusion of extraneous cells as result of using whole tissue could reduce the recovery of rare B-cell clones. In addition, some bias might be introduced because of cell subpopulation stability and frequency in whole spleen tissue [56, 57]. Nevertheless, in spite of the limitations of our methodology, it appears that the repertoire we detected correlated with mouse studies that have used selection and amplification methods of various kinds. For example, Collins *et al*. detected five of the same VH genes that we detected among our highest 10 used VH-gene segments. JH2 was also the most frequently detected in both of our studies [33]. Yang and Kaplinski detected V-gene segment use that paralleled our findings with V1-26 identified by them as the most frequently used [11, 42].

Few studies have explored the light chain repertoire; however, more characterization will be possible with increasing use of single cell amplification [58–60]. While strain specificity has been reported [33, 36], many Vκ-gene segments that were represented over one-percent of the time in unimmunized BALB/c mice were also identified in our study [26]. Aoki-Ota *et al*. also noted Vκ-gene segment skewing in their assessment of unimmunized C57BL/6 mice [45]. These similarities also suggest that the lack of amplification did not dramatically affect our assessment of the B-cell repertoire, and the differences seen are likely due to mouse-to-mouse variation that still manifests in our pooled samples.

In conclusion, we have presented an unamplified view of the conventionally housed, unimmunized, antibody repertoire. It appears that an RNASeq approach without amplification can provide an accurate assessment of V-gene use as well as a snapshot of the CDR3 present in the population; and helps validate this as a reasonable scientific approach. In addition, we lay the foundation for future work in our lab to characterize the unamplified whole tissue repertoire of the immunized C57BL/6 mouse.

## Supporting information

S1 Fig **V gene segment rankings among the three mouse pools for both IgH (A) and Igκ (B).** The most abundant gene segment is ranked as one. Dark red indicates higher rank moving to blue, of lower rank.
(PDF)

S2 Fig **CDR3 length for IgH (A) and Igκ (B) by pool.** The percent of repertoire CDR3 lengths from each mouse pools are displayed.
(TIF)

S3 Fig **Rankings of CDR3 sequences shared by all three mouse pools were uniform in both IgH (A) and Igκ (B).** The most abundant CDR3 sequence is ranked as one. Dark red indicates higher rank moving to blue, of lower rank.
(PDF)

S4 Fig Full CDR3 nucleotide alignment of IgH gene combination examined in Fig 7 (IGHV1-26, IGHD1-1, IGHJ1). (TIF)

## References

[1] Shahaf G, Barak M, Zuckerman NS, Swerdlin N, Gorfine M, Mehr R. Antigen-driven selection in germinal centers as reflected by the shape characteristics of immunoglobulin gene lineage trees: a large-scale simulation study. J Theor Biol. 2008;255(2):210–22. Epub 2008/09/13. doi: 10.1016/j.jtbi.2008.08.005 . 18786548 10.1016/j.jtbi.2008.08.005

[2] Cory S. Masterminding B Cells. J Immunol. 2015;195(3):763–5. Epub 2015/07/19. doi: 10.4049/jimmunol.1501277 . 26188069 10.4049/jimmunol.1501277

[3] Hozumi N, Tonegawa S. Evidence for somatic rearrangement of immunoglobulin genes coding for variable and constant regions. Proc Natl Acad Sci U S A. 1976;73(10):3628–32. Epub 1976/10/01. ; PubMed Central PMCID: PMCPMC431171. 824647 10.1073/pnas.73.10.3628 PMC431171

[4] Tonegawa S. Somatic generation of antibody diversity. Nature. 1983;302(5909):575–81. Epub 1983/04/14. . 6300689 10.1038/302575a0

[5] Early P, Huang H, Davis M, Calame K, Hood L. An immunoglobulin heavy chain variable region gene is generated from three segments of DNA: VH, D and JH. Cell. 1980;19(4):981–92. Epub 1980/04/01. . 6769593 10.1016/0092-8674(80)90089-6

[6] Tonegawa S. Reiteration frequency of immunoglobulin light chain genes: further evidence for somatic generation of antibody diversity. Proc Natl Acad Sci U S A. 1976;73(1):203–7. Epub 1976/01/01. ; PubMed Central PMCID: PMCPMC335869. 813222 10.1073/pnas.73.1.203 PMC335869

[7] Kabat EA, Wu TT, Bilofsky H. Evidence supporting somatic assembly of the DNA segments (minigenes), coding for the framework, and complementarity-determining segments of immunoglobulin variable regions. J Exp Med. 1979;149(6):1299–313. Epub 1979/06/01. ; PubMed Central PMCID: PMCPMC2184887. 109566 10.1084/jem.149.6.1299 PMC2184887

[8] Xu Z, Zan H, Pone EJ, Mai T, Casali P. Immunoglobulin class-switch DNA recombination: induction, targeting and beyond. Nat Rev Immunol. 2012;12(7):517–31. Epub 2012/06/26. doi: 10.1038/nri3216 ; PubMed Central PMCID: PMCPMC3545482. 22728528 10.1038/nri3216 PMC3545482

[9] Ippolito GC, Schelonka RL, Zemlin M, Ivanov II, Kobayashi R, Zemlin C, et al Forced usage of positively charged amino acids in immunoglobulin CDR-H3 impairs B cell development and antibody production. J Exp Med. 2006;203(6):1567–78. Epub 2006/06/07. doi: 10.1084/jem.20052217 ; PubMed Central PMCID: PMCPMC3212734. 16754718 10.1084/jem.20052217 PMC3212734

[10] Greiff V, Miho E, Menzel U, Reddy ST. Bioinformatic and Statistical Analysis of Adaptive Immune Repertoires. Trends Immunol. 2015;36(11):738–49. doi: 10.1016/j.it.2015.09.006 . 26508293 10.1016/j.it.2015.09.006

[11] Yang Y, Wang C, Yang Q, Kantor AB, Chu H, Ghosn EE, et al Distinct mechanisms define murine B cell lineage immunoglobulin heavy chain (IgH) repertoires. eLife. 2015;4:e09083 Epub 2015/10/01. doi: 10.7554/eLife.09083 ; PubMed Central PMCID: PMCPMC4714975. 26422511 10.7554/eLife.09083 PMC4714975

[12] Jiang N, Weinstein JA, Penland L, White RA 3rd, Fisher DS, Quake SR. Determinism and stochasticity during maturation of the zebrafish antibody repertoire. Proc Natl Acad Sci U S A. 2011;108(13):5348–53. Epub 2011/03/12. doi: 10.1073/pnas.1014277108 ; PubMed Central PMCID: PMCPMC3069157. 21393572 10.1073/pnas.1014277108 PMC3069157

[13] Racanelli V, Sansonno D, Piccoli C, D'Amore FP, Tucci FA, Dammacco F. Molecular characterization of B cell clonal expansions in the liver of chronically hepatitis C virus-infected patients. J Immunol. 2001;167(1):21–9. Epub 2001/06/22. . 11418627 10.4049/jimmunol.167.1.21

[14] Parameswaran P, Liu Y, Roskin KM, Jackson KK, Dixit VP, Lee JY, et al Convergent antibody signatures in human dengue. Cell Host Microbe. 2013;13(6):691–700. Epub 2013/06/19. doi: 10.1016/j.chom.2013.05.008 ; PubMed Central PMCID: PMCPMC4136508. 23768493 10.1016/j.chom.2013.05.008 PMC4136508

[15] Reddy ST, Ge X, Miklos AE, Hughes RA, Kang SH, Hoi KH, et al Monoclonal antibodies isolated without screening by analyzing the variable-gene repertoire of plasma cells. Nat Biotechnol. 2010;28(9):965–9. Epub 2010/08/31. doi: 10.1038/nbt.1673 . 20802495 10.1038/nbt.1673

[16] Gray SA, Moore M, VandenEkart EJ, Roque RP, Bowen RA, Van Hoeven N, et al Selection of therapeutic H5N1 monoclonal antibodies following IgVH repertoire analysis in mice. Antiviral Res. 2016;131:100–8. Epub 2016/04/26. doi: 10.1016/j.antiviral.2016.04.001 ; PubMed Central PMCID: PMCPMC4934617. 27109194 10.1016/j.antiviral.2016.04.001 PMC4934617

[17] Galson JD, Pollard AJ, Truck J, Kelly DF. Studying the antibody repertoire after vaccination: practical applications. Trends Immunol. 2014;35(7):319–31. Epub 2014/05/27. doi: 10.1016/j.it.2014.04.005 . 24856924 10.1016/j.it.2014.04.005

[18] Ademokun A, Wu YC, Martin V, Mitra R, Sack U, Baxendale H, et al Vaccination-induced changes in human B-cell repertoire and pneumococcal IgM and IgA antibody at different ages. Aging Cell. 2011;10(6):922–30. Epub 2011/07/06. doi: 10.1111/j.1474-9726.2011.00732.x ; PubMed Central PMCID: PMCPMC3264704. 21726404 10.1111/j.1474-9726.2011.00732.x PMC3264704

[19] Rosenquist R, Thunberg U, Li AH, Forestier E, Lonnerholm G, Lindh J, et al Clonal evolution as judged by immunoglobulin heavy chain gene rearrangements in relapsing precursor-B acute lymphoblastic leukemia. Eur J Haematol. 1999;63(3):171–9. Epub 1999/09/15. . 10485272 10.1111/j.1600-0609.1999.tb01765.x

[20] Bashford-Rogers RJ, Palser AL, Huntly BJ, Rance R, Vassiliou GS, Follows GA, et al Network properties derived from deep sequencing of human B-cell receptor repertoires delineate B-cell populations. Genome Res. 2013;23(11):1874–84. Epub 2013/06/08. doi: 10.1101/gr.154815.113 ; PubMed Central PMCID: PMCPMC3814887. 23742949 10.1101/gr.154815.113 PMC3814887

[21] Bashford-Rogers RJ, Nicolaou KA, Bartram J, Goulden NJ, Loizou L, Koumas L, et al Eye on the B-ALL: B-cell receptor repertoires reveal persistence of numerous B-lymphoblastic leukemia subclones from diagnosis to relapse. Leukemia. 2016;30(12):2312–21. Epub 2016/05/24. doi: 10.1038/leu.2016.142 ; PubMed Central PMCID: PMCPMC5155029. 27211266 10.1038/leu.2016.142 PMC5155029

[22] van Belzen N, Hupkes PE, Doekharan D, Hoogeveen-Westerveld M, Dorssers LC, van't Veer MB. Detection of minimal disease using rearranged immunoglobulin heavy chain genes from intermediate- and high-grade malignant B cell non-Hodgkins lymphoma. Leukemia. 1997;11(10):1742–52. Epub 1997/10/27. . 9324296 10.1038/sj.leu.2400797

[23] Zuckerman NS, Howard WA, Bismuth J, Gibson K, Edelman H, Berrih-Aknin S, et al Ectopic GC in the thymus of myasthenia gravis patients show characteristics of normal GC. Eur J Immunol. 2010;40(4):1150–61. Epub 2010/01/28. doi: 10.1002/eji.200939914 . 20104489 10.1002/eji.200939914

[24] Tan YC, Kongpachith S, Blum LK, Ju CH, Lahey LJ, Lu DR, et al Barcode-enabled sequencing of plasmablast antibody repertoires in rheumatoid arthritis. Arthritis Rheumatol. 2014;66(10):2706–15. Epub 2014/06/27. doi: 10.1002/art.38754 ; PubMed Central PMCID: PMCPMC4560105. 24965753 10.1002/art.38754 PMC4560105

[25] Greiff V, Bhat P, Cook SC, Menzel U, Kang W, Reddy ST. A bioinformatic framework for immune repertoire diversity profiling enables detection of immunological status. Genome Med. 2015;7(1):49 Epub 2015/07/04. doi: 10.1186/s13073-015-0169-8 ; PubMed Central PMCID: PMCPMC4489130. 26140055 10.1186/s13073-015-0169-8 PMC4489130

[26] Lu J, Panavas T, Thys K, Aerssens J, Naso M, Fisher J, et al IgG variable region and VH CDR3 diversity in unimmunized mice analyzed by massively parallel sequencing. Mol Immunol. 2014;57(2):274–83. doi: 10.1016/j.molimm.2013.09.008 . 24211535 10.1016/j.molimm.2013.09.008

[27] Briney BS, Willis JR, McKinney BA, Crowe JE, Jr. High-throughput antibody sequencing reveals genetic evidence of global regulation of the naive and memory repertoires that extends across individuals. Genes Immun. 2012;13(6):469–73. Epub 2012/05/25. doi: 10.1038/gene.2012.20 . 22622198 10.1038/gene.2012.20

[28] Boyd SD, Gaeta BA, Jackson KJ, Fire AZ, Marshall EL, Merker JD, et al Individual variation in the germline Ig gene repertoire inferred from variable region gene rearrangements. J Immunol. 2010;184(12):6986–92. Epub 2010/05/25. doi: 10.4049/jimmunol.1000445 ; PubMed Central PMCID: PMCPMC4281569. 20495067 10.4049/jimmunol.1000445 PMC4281569

[29] Watson CT, Steinberg KM, Huddleston J, Warren RL, Malig M, Schein J, et al Complete haplotype sequence of the human immunoglobulin heavy-chain variable, diversity, and joining genes and characterization of allelic and copy-number variation. Am J Hum Genet. 2013;92(4):530–46. Epub 2013/04/02. doi: 10.1016/j.ajhg.2013.03.004 ; PubMed Central PMCID: PMCPMC3617388. 23541343 10.1016/j.ajhg.2013.03.004 PMC3617388

[30] Sasso EH, Van Dijk KW, Milner EC. Prevalence and polymorphism of human VH3 genes. J Immunol. 1990;145(8):2751–7. Epub 1990/10/15. . 1976703

[31] Milner EC, Hufnagle WO, Glas AM, Suzuki I, Alexander C. Polymorphism and utilization of human VH Genes. Ann N Y Acad Sci. 1995;764:50–61. Epub 1995/09/29. . 7486575 10.1111/j.1749-6632.1995.tb55806.x

[32] Wang Y, Jackson KJ, Gaeta B, Pomat W, Siba P, Sewell WA, et al Genomic screening by 454 pyrosequencing identifies a new human IGHV gene and sixteen other new IGHV allelic variants. Immunogenetics. 2011;63(5):259–65. Epub 2011/01/21. doi: 10.1007/s00251-010-0510-8 . 21249354 10.1007/s00251-010-0510-8

[33] Collins AM, Wang Y, Roskin KM, Marquis CP, Jackson KJ. The mouse antibody heavy chain repertoire is germline-focused and highly variable between inbred strains. Philos Trans R Soc Lond B Biol Sci. 2015;370(1676). Epub 2015/07/22. doi: 10.1098/rstb.2014.0236 ; PubMed Central PMCID: PMCPMC4528413. 26194750 10.1098/rstb.2014.0236 PMC4528413

[34] Georgiou G, Ippolito GC, Beausang J, Busse CE, Wardemann H, Quake SR. The promise and challenge of high-throughput sequencing of the antibody repertoire. Nat Biotechnol. 2014;32(2):158–68. doi: 10.1038/nbt.2782 ; PubMed Central PMCID: PMC4113560. 24441474 10.1038/nbt.2782 PMC4113560

[35] Rettig TA, Ward C., Pecaut M.J., Chapes S.K. Validation of Methods to Assess the Immunoglobulin Gene Repertoire in Tissues Obtained from Mice on the International Space Station. Gravit Space Res. 2017;5(1):2–23. 29270444 PMC5736159

[36] Greiff V, Menzel U, Miho E, Weber C, Riedel R, Cook S, et al Systems Analysis Reveals High Genetic and Antigen-Driven Predetermination of Antibody Repertoires throughout B Cell Development. Cell Rep. 2017;19(7):1467–78. Epub 2017/05/18. doi: 10.1016/j.celrep.2017.04.054 . 28514665 10.1016/j.celrep.2017.04.054

[37] Huerkamp MJ. It's in the bag: Easy and medically sound rodent gas anesthesia induction. Tech Talk. 2000;5(3):3.

[38] Alamyar E, Duroux P, Lefranc MP, Giudicelli V. IMGT((R)) tools for the nucleotide analysis of immunoglobulin (IG) and T cell receptor (TR) V-(D)-J repertoires, polymorphisms, and IG mutations: IMGT/V-QUEST and IMGT/HighV-QUEST for NGS. Methods Mol Biol. 2012;882:569–604. Epub 2012/06/06. doi: 10.1007/978-1-61779-842-9_32 . 22665256 10.1007/978-1-61779-842-9_32

[39] Katoh K, Misawa K, Kuma K, Miyata T. MAFFT: a novel method for rapid multiple sequence alignment based on fast Fourier transform. Nucleic Acids Res. 2002;30(14):3059–66. Epub 2002/07/24. ; PubMed Central PMCID: PMCPMC135756. 12136088 10.1093/nar/gkf436 PMC135756

[40] Krzywinski M, Schein J, Birol I, Connors J, Gascoyne R, Horsman D, et al Circos: an information aesthetic for comparative genomics. Genome Res. 2009;19(9):1639–45. Epub 2009/06/23. doi: 10.1101/gr.092759.109 ; PubMed Central PMCID: PMCPMC2752132. 19541911 10.1101/gr.092759.109 PMC2752132

[41] de Bono B, Madera M, Chothia C. VH gene segments in the mouse and human genomes. J Mol Biol. 2004;342(1):131–43. Epub 2004/08/18. doi: 10.1016/j.jmb.2004.06.055 . 15313612 10.1016/j.jmb.2004.06.055

[42] Kaplinsky J, Li A, Sun A, Coffre M, Koralov SB, Arnaout R. Antibody repertoire deep sequencing reveals antigen-independent selection in maturing B cells. Proc Natl Acad Sci U S A. 2014;111(25):E2622–9. doi: 10.1073/pnas.1403278111 ; PubMed Central PMCID: PMC4078805. 24927543 10.1073/pnas.1403278111 PMC4078805

[43] Kono N, Sun L, Toh H, Shimizu T, Xue H, Numata O, et al Deciphering antigen-responding antibody repertoires by using next-generation sequencing and confirming them through antibody-gene synthesis. Biochem Biophys Res Commun. 2017;487(2):300–6. Epub 2017/04/17. doi: 10.1016/j.bbrc.2017.04.054 . 28412367 10.1016/j.bbrc.2017.04.054

[44] Greiff V, Menzel U, Haessler U, Cook SC, Friedensohn S, Khan TA, et al Quantitative assessment of the robustness of next-generation sequencing of antibody variable gene repertoires from immunized mice. BMC Immunol. 2014;15:40 Epub 2014/10/17. doi: 10.1186/s12865-014-0040-5 ; PubMed Central PMCID: PMCPMC4233042. 25318652 10.1186/s12865-014-0040-5 PMC4233042

[45] Aoki-Ota M, Torkamani A, Ota T, Schork N, Nemazee D. Skewed primary Igkappa repertoire and V-J joining in C57BL/6 mice: implications for recombination accessibility and receptor editing. J Immunol. 2012;188(5):2305–15. Epub 2012/01/31. doi: 10.4049/jimmunol.1103484 ; PubMed Central PMCID: PMCPMC3288532. 22287713 10.4049/jimmunol.1103484 PMC3288532

[46] Choi NM, Loguercio S, Verma-Gaur J, Degner SC, Torkamani A, Su AI, et al Deep sequencing of the murine IgH repertoire reveals complex regulation of nonrandom V gene rearrangement frequencies. J Immunol. 2013;191(5):2393–402. Epub 2013/07/31. doi: 10.4049/jimmunol.1301279 ; PubMed Central PMCID: PMCPMC3778908. 23898036 10.4049/jimmunol.1301279 PMC3778908

[47] Klein-Schneegans AS, Kuntz L, Fonteneau P, Loor F. Serum concentrations of IgM, IgG1, IgG2b, IgG3 and IgA in C57BL/6 mice and their congenics at the lpr (lymphoproliferation) locus. J Autoimmun. 1989;2(6):869–75. Epub 1989/12/01. . 2619870 10.1016/0896-8411(89)90013-9

[48] Glanville J, Kuo TC, von Budingen HC, Guey L, Berka J, Sundar PD, et al Naive antibody gene-segment frequencies are heritable and unaltered by chronic lymphocyte ablation. Proc Natl Acad Sci U S A. 2011;108(50):20066–71. Epub 2011/11/30. doi: 10.1073/pnas.1107498108 ; PubMed Central PMCID: PMCPMC3250199. 22123975 10.1073/pnas.1107498108 PMC3250199

[49] Chao A, Tsay PK, Lin SH, Shau WY, Chao DY. The applications of capture-recapture models to epidemiological data. Stat Med. 2001;20(20):3123–57. Epub 2001/10/09. . 11590637 10.1002/sim.996

[50] Chapman DG, University of California B. Some properties of the hypergeometric distribution with applications to zoological sample censuses Berkeley: University of California Press; 1951 131–59 p. p.

[51] Calis JJ, Rosenberg BR. Characterizing immune repertoires by high throughput sequencing: strategies and applications. Trends Immunol. 2014;35(12):581–90. Epub 2014/10/13. doi: 10.1016/j.it.2014.09.004 ; PubMed Central PMCID: PMCPMC4390416. 25306219 10.1016/j.it.2014.09.004 PMC4390416

[52] Kunik V, Peters B, Ofran Y. Structural consensus among antibodies defines the antigen binding site. PLoS Comput Biol. 2012;8(2):e1002388 Epub 2012/03/03. doi: 10.1371/journal.pcbi.1002388 ; PubMed Central PMCID: PMCPMC3285572. 22383868 10.1371/journal.pcbi.1002388 PMC3285572

[53] Sela-Culang I, Kunik V, Ofran Y. The structural basis of antibody-antigen recognition. Front Immunol. 2013;4:302 Epub 2013/10/12. doi: 10.3389/fimmu.2013.00302 ; PubMed Central PMCID: PMCPMC3792396. 24115948 10.3389/fimmu.2013.00302 PMC3792396

[54] Banga S, Coursen JD, Portugal S, Tran TM, Hancox L, Ongoiba A, et al Impact of acute malaria on pre-existing antibodies to viral and vaccine antigens in mice and humans. PloS One. 2015;10(4):e0125090 Epub 2015/04/29. doi: 10.1371/journal.pone.0125090 ; PubMed Central PMCID: PMCPMC4412709. 25919588 10.1371/journal.pone.0125090 PMC4412709

[55] Boyd SD, Marshall EL, Merker JD, Maniar JM, Zhang LN, Sahaf B, et al Measurement and clinical monitoring of human lymphocyte clonality by massively parallel VDJ pyrosequencing. Sci Transl Med. 2009;1(12):12ra23 Epub 2010/02/18. ; PubMed Central PMCID: PMC2819115. 20161664 10.1126/scitranslmed.3000540 PMC2819115

[56] Allman DM, Ferguson SE, Lentz VM, Cancro MP. Peripheral B cell maturation. II. Heat-stable antigen(hi) splenic B cells are an immature developmental intermediate in the production of long-lived marrow-derived B cells. J Immunol. 1993;151(9):4431–44. Epub 1993/11/01. . 8409411

[57] Allman D, Lindsley RC, DeMuth W, Rudd K, Shinton SA, Hardy RR. Resolution of three nonproliferative immature splenic B cell subsets reveals multiple selection points during peripheral B cell maturation. J Immunol. 2001;167(12):6834–40. Epub 2001/12/12. . 11739500 10.4049/jimmunol.167.12.6834

[58] DeKosky BJ, Ippolito GC, Deschner RP, Lavinder JJ, Wine Y, Rawlings BM, et al High-throughput sequencing of the paired human immunoglobulin heavy and light chain repertoire. Nat Biotechnol. 2013;31(2):166–9. Epub 2013/01/22. doi: 10.1038/nbt.2492 ; PubMed Central PMCID: PMCPMC3910347. 23334449 10.1038/nbt.2492 PMC3910347

[59] DeKosky BJ, Kojima T, Rodin A, Charab W, Ippolito GC, Ellington AD, et al In-depth determination and analysis of the human paired heavy- and light-chain antibody repertoire. Nature Med. 2015;21(1):86–91. Epub 2014/12/17. doi: 10.1038/nm.3743 . 25501908 10.1038/nm.3743

[60] Busse CE, Czogiel I, Braun P, Arndt PF, Wardemann H. Single-cell based high-throughput sequencing of full-length immunoglobulin heavy and light chain genes. Eur J Immunol. 2014;44(2):597–603. Epub 2013/10/12. doi: 10.1002/eji.201343917 . 24114719 10.1002/eji.201343917

[61] Ward C, Rettig T, Hlavacek S, Bye B, Pecaut MJ, Chapes SK. Effects of spaceflight on the immunoglobulin repertoire of unimmunized C57BL/6 mice. Life Sciences in Space Res. 2018; 16:63–75. https://doi.org/10.1016/j.lssr.2017.11.003. 10.1016/j.lssr.2017.11.003 PMC5826609 29475521

