# Supplementary material for: Characterization of the naive murine antibody repertoire using unamplified high-throughput sequencing
Source: PLoS One. 2018 Jan 10;13(1):e0190982. doi: 10.1371/journal.pone.0190982 (PMC5761896; doi:10.1371/journal.pone.0190982)
Supplement: S1 Fig — V gene segment rankings among the three mouse pools for both IgH (A) and Igκ (B). The most abundant gene segment is ranked as one. Dark red indicates higher rank moving to blue, of lower rank. (PDF) [file pone.0190982.s001.pdf]

A

	Pool 1	Pool 2	Pool 3
V1-80*01	7.83	7.00	3.34
V1-26*01	4.29	3.96	3.86
V6-3*01	2.91	2.74	4.93
V1-53*01	3.80	2.96	2.73
V1-55*01	3.57	2.40	3.34
V3-6*01	2.68	2.28	3.81
V1-9*01	3.00	3.54	1.85
V1-18*01	4.05	3.09	1.21
V9-3*01	2.46	2.72	2.46
V1-50*01	1.43	2.32	3.89
V1-64*01	1.88	2.08	3.64
V6-6*01	2.72	2.22	1.51
V1-82*01	2.06	1.97	1.96
V8-8*01	2.22	1.74	1.94
V1-78*01	2.66	1.17	1.51
V1-81*01	1.76	1.77	1.24
V7-3*01	1.68	1.64	1.45
V1-72*01	1.32	1.41	1.90
V10-1*01	1.62	1.62	1.37
V1-22*01	1.50	1.77	1.24
V5-17*01	1.38	1.75	1.37
V2-2*01	1.16	1.75	1.40
V14-4*01	1.50	1.30	1.43
V1-76*01	0.87	2.22	1.11
V2-3*01	1.54	1.40	1.24
V4-1*01	1.09	2.05	1.02
V1S92*01	1.43	0.99	1.49
V11-2*01	1.59	1.39	0.86
V1S95*01	1.35	0.97	1.50
V1-69*01	0.86	1.40	1.40
V1-39*01	1.04	0.85	1.36
V1-52*01	0.77	0.98	1.46
V1-15*01	1.05	0.96	1.06
V10-3*01	0.78	1.47	0.80
V2-9*01	1.39	0.82	0.83
V1-75*01	1.30	0.72	0.89
V2-9-1*01	0.93	0.78	1.13
V1-19*01	0.74	1.07	1.01
V14-2*01	1.03	0.99	0.80
V8-12*01	1.03	1.00	0.78
V1-66*01	0.35	0.47	1.92
V1-59*01	0.91	0.53	1.20
V5-4*01	0.99	0.91	0.65
V5-16*01	0.89	0.83	0.74
V1-7*01	0.80	0.88	0.76
V1S108*01	0.13	0.74	1.57
V5-9-1*02	0.31	0.30	1.74
V1-74*01	0.45	0.62	1.18
V2-6*01	0.02	1.98	0.24
V2-6-8*01	0.02	1.98	0.23
V9-1*01	0.43	0.79	0.93
V1-63*01	1.69	0.23	0.17
V1-42*01	0.77	0.44	0.80
V9-4*01	1.06	0.52	0.41
V1-61*01	0.44	0.60	0.87
V3-1*01	0.92	0.44	0.51
V1-54*01	0.82	0.47	0.59
V14-3*01	0.60	0.58	0.68
V1-4*01	0.56	0.68	0.43
V2-5*01	0.39	0.67	0.54
V3-8*01	0.45	0.67	0.41
V1-12*01	0.54	0.62	0.34
V1-85*01	0.67	0.30	0.45

	Pool 1	Pool 2	Pool 3
V5-6*01	0.39	0.37	0.54
V1-5*01	0.25	0.51	0.41
V1-84*01	0.50	0.26	0.41
V8-5*01	0.43	0.32	0.39
V13-2*01	0.59	0.24	0.30
V14-1*01	0.32	0.52	0.28
V9-2*01	0.14	0.32	0.56
V5S21*01	0.07	0.11	0.70
V1-47*01	0.34	0.28	0.25
V1-58*01	0.35	0.29	0.21
V1-34*01	0.30	0.22	0.31
V3-5*01	0.27	0.33	0.21
V2-4*01	0.27	0.39	0.15
V1-20*01	0.19	0.31	0.24
V12-3*01	0.30	0.25	0.16
V5-12*01	0.17	0.17	0.32
V1-77*01	0.20	0.19	0.19
V1S96*01	0.01	0.18	0.36
V1-62-2*01	0.13	0.10	0.32
V1-71*01	0.13	0.10	0.32
V5-9*01	0.23	0.17	0.14
V1-36*01	0.16	0.23	0.15
V1-11*01	0.11	0.23	0.08
V1-62-3*01	0.02	0.02	0.39
V5-15*01	0.07	0.23	0.08
V5-2*01	0.15	0.12	0.10
V8-2*01	0.09	0.14	0.12
V1S5*01	0.00	0.04	0.29
V8S9*01	0.04	0.13	0.15
V1-67*01	0.07	0.14	0.10
V7-4*01	0.18	0.07	0.06
V3-4*01	0.14	0.08	0.08
V1S87*01	0.00	0.11	0.17
V1S103*01	0.00	0.06	0.21
V1S65*01	0.06	0.07	0.13
V15-2*01	0.07	0.07	0.10
V1-49*01	0.05	0.09	0.11
V1-31*01	0.08	0.07	0.08
V1-56*01	0.03	0.04	0.16
V1S107*01	0.04	0.07	0.10
V11-1*01	0.01	0.12	0.05
V3-3*01	0.09	0.07	0.03
V1S100*01	0.02	0.04	0.10
V1-43*01	0.04	0.05	0.06
V1-23*01	0.05	0.04	0.06
V8-11*01	0.01	0.05	0.08
V2-7*01	0.04	0.06	0.04
V1-37*01	0.06	0.04	0.03
V16-1*01	0.03	0.05	0.05
V6-7*01	0.04	0.02	0.05
V1S67*01	0.01	0.03	0.07
V1S68*01	0.01	0.03	0.06
V7-2*01	0.03	0.04	0.03
V3S7*01	0.01	0.03	0.04
V8-6*01	0.00	0.02	0.05
V8-4*01	0.02	0.03	0.02
V6-4*01	0.01	0.02	0.03
V8-9*01	0.02	0.01	0.02
V1-62-1*01	0.00	0.01	0.04
V1-14*01	0.01	0.02	0.02
V8-8-1*01	0.01	0.02	0.02
V5-12-4*01	0.01	0.01	0.02
V8-13*01	0.00	0.00	0.04

	Pool 1	Pool 2	Pool 3
V5-9*04	0.00	0.01	0.02
V6-5*01	0.01	0.01	0.01
V13-1*01	0.01	0.01	0.01
V1-17-1*01	0.01	0.01	0.00
V5S24*01	0.01	0.01	0.00
V1-24*01	0.00	0.00	0.00

B

	Pool 1	Pool 2	Pool 3
V1-110*01	4.80	13.44	6.15
V1-117*01	8.13	5.30	7.09
V5-39*01	10.33	1.03	2.84
V10-96*01	2.69	5.04	4.77
V4-55*01	4.20	3.87	3.62
V1-135*01	3.62	2.50	5.33
V3-4*01	3.40	2.11	2.71
V5-43*01	3.25	1.71	1.53
V10-94*01	0.97	3.45	2.04
V6-15*01	1.50	1.84	3.00
V14-111*01	2.02	2.28	1.97
V12-44*01	1.67	3.09	1.41
V3-2*01	2.24	1.23	2.69
V19-93*01	2.17	2.36	1.63
V2-137*01	1.99	1.44	2.50
V12-46*01	2.22	2.33	1.35
V8-30*01	1.60	1.85	2.40
V4-59*01	1.92	1.59	1.98
V8-24*01	2.25	1.57	1.35
V9-120*01	1.76	1.58	1.70
V4-72*01	2.05	1.08	1.21
V6-23*01	1.04	1.68	1.59
V6-17*01	1.44	1.55	1.28
V14-126*01	1.63	1.38	1.22
V4-70*01	0.65	1.12	2.37
V15-103*01	1.02	1.87	1.23
V8-27*01	1.39	1.33	1.09
V4-57*01	2.20	0.65	0.78
V5-48*01	1.10	0.92	1.21
V16-104*01	1.02	0.96	1.15
V2-109*01	0.99	0.94	0.90
V17-127*01	1.06	0.82	0.92
V6-32*01	0.72	1.06	0.92
V4-53*01	1.01	1.02	0.63
V8-21*01	0.38	0.61	1.66
V8-19*01	0.86	0.92	0.81
V6-25*01	0.73	0.63	1.23
V17-121*01	0.97	0.68	0.80
V4-68*01	0.58	1.04	0.82
V9-124*01	0.83	0.95	0.62
V4-86*01	0.53	1.07	0.80
V12-89*01	0.96	0.65	0.75
V12-41*01	0.66	0.58	1.06
V4-91*01	0.68	0.58	0.78
V3-5*01	0.65	0.54	0.80
V4-63*01	0.45	1.30	0.22
V3-12*01	0.41	0.78	0.74
V3-10*01	0.54	0.55	0.81
V2-112*01	0.40	1.05	0.40
V8-28*01	0.68	0.49	0.51
V6-20*01	0.53	0.72	0.38
V4-57-1*01	0.41	0.57	0.54
V14-100*01	0.54	0.51	0.46
V6-13*01	0.30	0.26	0.81
V1-122*01	0.51	0.32	0.50
V5-45*01	0.60	0.31	0.39
V3-7*01	0.27	0.55	0.38
V4-61*01	0.61	0.29	0.31
V13-85*01	0.28	0.36	0.50
V8-16*01	0.28	0.49	0.32
V1-99*01	0.61	0.22	0.25
V4-79*01	0.20	0.23	0.64
V13-84*01	0.29	0.41	0.36

	Pool 1	Pool 2	Pool 3
V12-98*01	0.23	0.47	0.29
V4-71*01	0.03	0.39	0.55
V4-74*01	0.34	0.33	0.28
V4-80*01	0.23	0.32	0.36
V6-14*01	0.38	0.22	0.29
V4-50*01	0.32	0.26	0.30
V1-88*01	0.26	0.35	0.25
V7-33*01	0.09	0.25	0.28
V4-73*01	0.03	0.28	0.31
V11-125*01	0.10	0.23	0.26
V6-32*02	0.11	0.27	0.20
V4-58*01	0.15	0.19	0.21
V1-133*01	0.14	0.12	0.26
V4-69*01	0.11	0.14	0.27
V10-95*01	0.12	0.17	0.16
V9-129*01	0.11	0.20	0.14
V4-78*01	0.05	0.21	0.18
V3-1*01	0.09	0.17	0.18
V8-18*01	0.09	0.07	0.22
V12-38*01	0.11	0.10	0.15
V4-90*01	0.14	0.10	0.09
V8-23-1*01	0.08	0.13	0.10
V4-62*01	0.04	0.12	0.14
V8-34*01	0.05	0.12	0.12
V12-e*01	0.02	0.14	0.12
V4-81*01	0.09	0.11	0.08
V9-123*01	0.10	0.08	0.09
V14-130*01	0.08	0.08	0.07
V3-3*01	0.05	0.07	0.08
V4-51*01	0.06	0.07	0.06
V3-9*01	0.02	0.06	0.09
V1-131*01	0.01	0.06	0.10
V2-a*01	0.02	0.08	0.07
V4-52*01	0.03	0.05	0.08
V4-54*01	0.03	0.05	0.08
V4-92*01	0.07	0.05	0.04
V6-29*01	0.04	0.07	0.03
V1-132*01	0.05	0.03	0.06
V5-37*01	0.02	0.05	0.05
V18-36*01	0.04	0.04	0.03
V8-26*01	0.01	0.03	0.03
V20-101-2*01	0.01	0.01	0.02
V6-c*01	0.00	0.02	0.01
V6-d*01	0.01	0.01	0.00
V1-35*01	0.00	0.01	0.01
V6-b*01	0.00	0.01	0.01
