# Supplementary figures and images for: Characterization of the naive murine antibody repertoire using unamplified high-throughput sequencing
Source: PLoS One. 2018 Jan 10;13(1):e0190982. doi: 10.1371/journal.pone.0190982 (PMC5761896; doi:10.1371/journal.pone.0190982)

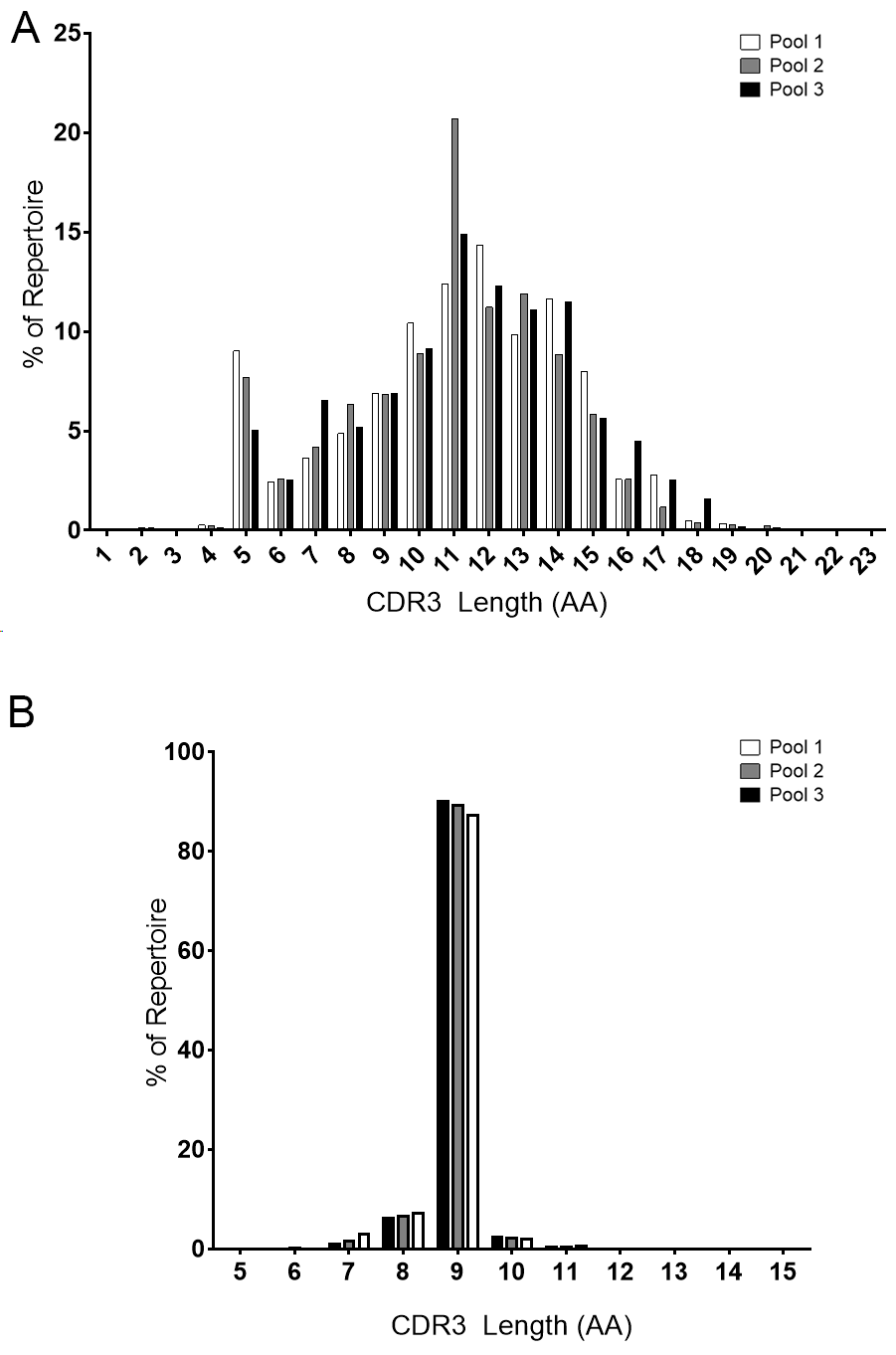

Supplement: S2 Fig — CDR3 length for IgH (A) and Igκ (B) by pool. The percent of repertoire CDR3 lengths from each mouse pools are displayed. (TIF) [file pone.0190982.s002.tif]

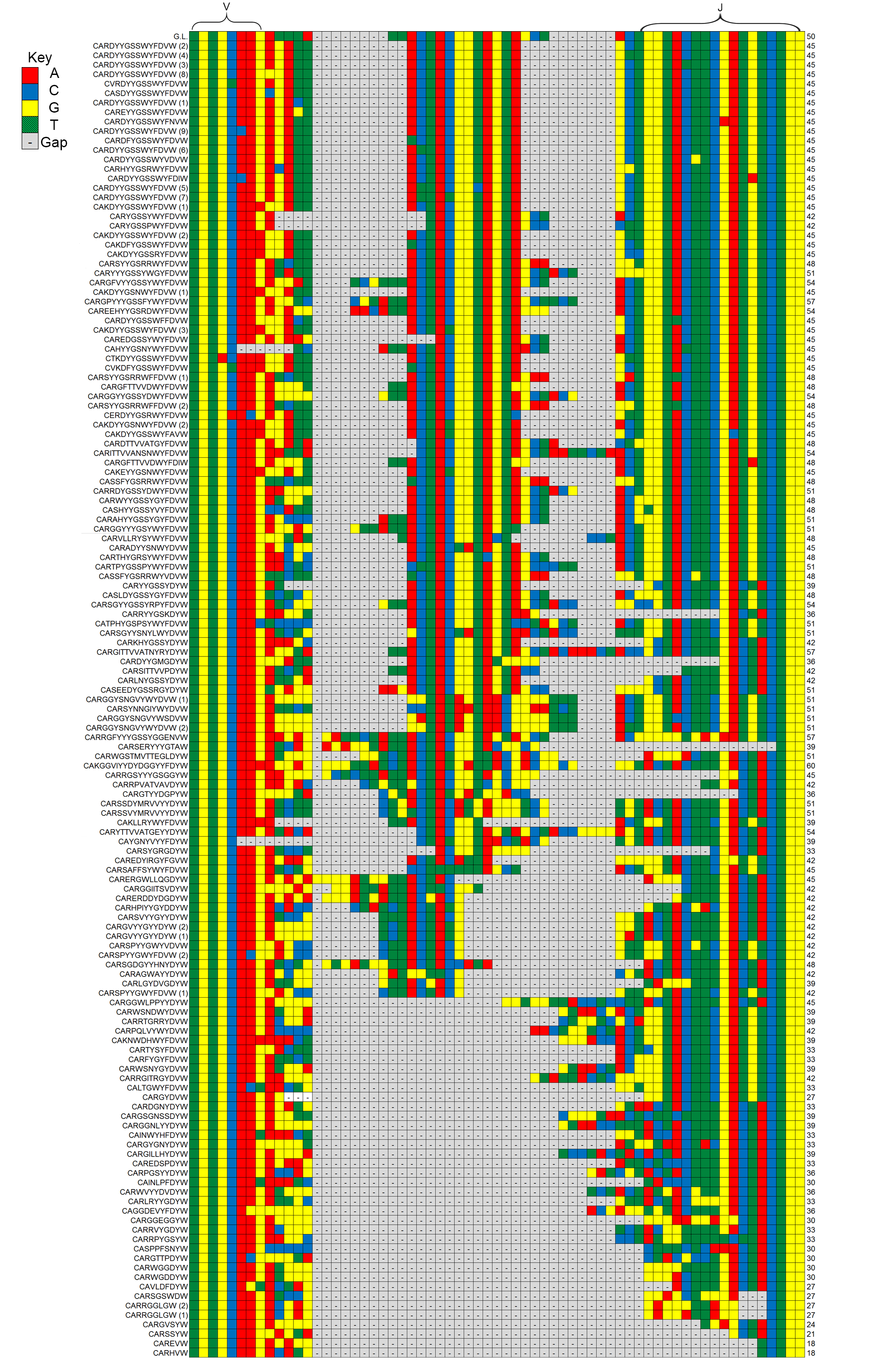

Supplement: S4 Fig — (TIF) [file pone.0190982.s004.tif]
