# Supplementary material for: Characterization of the naive murine antibody repertoire using unamplified high-throughput sequencing
Source: PLoS One. 2018 Jan 10;13(1):e0190982. doi: 10.1371/journal.pone.0190982 (PMC5761896; doi:10.1371/journal.pone.0190982)
Supplement: S3 Fig — Rankings of CDR3 sequences shared by all three mouse pools were uniform in both IgH (A) and Igκ (B). The most abundant CDR3 sequence is ranked as one. Dark red indicates higher rank moving to blue, of lower rank. (PDF) [file pone.0190982.s003.pdf]

A

	Pool 1	Pool 2	Pool 3
CARGAYW	1	2	3
CARDYYGSSWYFDVW	18	6	6
CMRYSNYWYFDVW	4	11	15
CARGGYW	16	5	18
CARGTYW	45	18	7
CMRYGNYWYFDVW	37	15	23
CARGYYFDYW	45	9	22
CARGDYW	9	26	45
CARDSNWYFDVW	39	34	19
CARGPYW	13	158	34
CARDNWDWYFDVW	189	20	55
CMRYSSYWYFDVW	189	14	62
CARW	125	73	170
CARGGFAYW	97	158	137
CAKKGYAMDYW	97	79	299
CARDYYGSSYWYFDVW	266	192	55
CARRLDYW	189	228	170
CMRYGSSYWYFDVW	266	26	299
CTTVRYW	125	301	170
CARPYDYW	28	301	299
CAQMRGFAYW	125	464	40
CARRFDYW	413	228	96
CMRYGSSWYFDVW	125	192	449
CARDDGYYWYFDVW	189	301	299
CARRYYGSSYWYFDVW	125	464	219
CTTLRYW	266	112	449
CAKNWDYW	189	464	219
CARDYDYWYFDVW	413	192	299
CARRDYGSSYWYFDVW	24	918	52
CARYGPYYFDYW	62	31	933
CARIYYYGSYWYFDVW	266	464	299
CARDEWFAYW	760	79	299
CARHYYGSSYWYFDVW	760	84	299
CARDWDYWYFDVW	266	464	449
CARGDGFDYW	97	192	933
CARHYGSSYWYFDVW	266	63	933
CARGYYW	760	57	449
CAKGDYGSSWFAYW	266	130	933
CARLYYGSSYWYFDVW	97	301	933
CTRGYYFDYW	760	158	449
CARGDGYYFDYW	760	464	219
CARGGDYW	760	464	219
CARGAMDYW	189	464	933
CARYDGYFDYW	413	301	933
CTVYYGSTWFAYW	413	301	933
CARRDYW	760	464	449
CARSYYFDYW	760	464	449
CAREGDYDYDWYFDVW	760	918	52
CARDYYGSSGYFDVW	17	918	933
CTRVAYW	760	192	933
CTRWDYW	760	192	933
CARYAMDYW	760	228	933
CARDYYGSSFDYW	760	918	299
CARYSNYFDYW	760	918	299
CANYGSSYWYFDVW	760	301	933
CARSLDYW	760	301	933
CASELGGFAYW	760	301	933
CASPNWDWYFDVW	760	301	933
CAREDYW	189	918	933
CARDGFAYW	266	918	933
CARKLDYW	760	464	933
CARNWDYAMDYW	760	464	933
CARYYYGSSYWYFDVW	760	464	933

	Pool 1	Pool 2	Pool 3
CARSYYFDYW	760	464	449
CAREGDYDYDWYFDVW	760	918	52
CARDYYGSSGYFDVW	17	918	933
CTRVAYW	760	192	933
CTRWDYW	760	192	933
CARYAMDYW	760	192	933
CARDYYGSSFDYW	760	918	299
CARYSNYFDYW	760	918	299
CANYGSSYWYFDVW	760	301	933
CARSLDYW	760	301	933
CASELGGFAYW	760	301	933
CASPNWDWYFDVW	760	301	933
CAREDYW	189	918	933
CARDGFAYW	266	918	933
CARKLDYW	760	464	933
CARNWDYAMDYW	760	464	933
CARYYYGSSYWYFDVW	760	464	933
CARSGTDYW	413	918	933
CARDFDYW	760	918	933
CARDGSSYWYFDVW	760	918	933
CARDYFDYW	760	918	933
CARESNYYFDYW	760	918	933
CARRDWYFDVW	760	918	933
CARSYYYAMDYW	760	918	933
CARYGNYAMDYW	760	918	933
CARYSNYAMDYW	760	918	933
CATGFAYW	760	918	933
CTGLYYFDYW	760	918	933
CTYYYGSSDFDYW	760	918	933

B

	Pool 1	Pool 2	Pool 3
CQQWSSYPLTF	13	6	2
CQQWSSNPPTF	2	18	7
CQQWSSYPFTF	6	3	24
CQQHYSTPLTF	7	12	16
CQQGNTLPWTF	21	7	12
CQQSNEDPRTF	11	21	10
CFQGSHVPWTF	17	12	18
CFQGSHVPYTF	16	9	22
CQQHYSTPYTF	24	14	13
CQQYSSYPLTF	26	15	11
CQQYNSYPLTF	33	15	5
CQQSNSWPLTF	20	26	8
CQNGHSFPLTF	1	55	1
CQQSNEDPYTF	14	25	25
CLQHGESPYTF	19	17	29
CLQYDEFPYTF	37	10	20
CSQSTHVPYTF	24	23	21
CLQYASSPYTF	21	24	26
CFQGSHVPLTF	5	30	38
CLQYDNLWTF	18	18	37
CSQSTHVPWTF	23	20	31
CQQYNSYPYTF	45	22	16
CMQHLEYPYTF	43	40	9
CQQWSSNPLTF	39	26	28
CQQHYSTPWTF	35	33	32
CQQSNSWPYTF	29	46	27
CQQWSSYPPTF	28	40	34
CQQGNTLPYTF	27	34	42
CQQSNSWPFTF	3	89	30
CQQWNYPLITF	94	8	22
CMQHLEYPFTF	30	43	59
CQQYSKLPWTF	82	4	52
CLQYDNLYTF	69	38	33
CQQGNTLPRTF	34	44	63
CLQSDNMPLTF	45	53	44
CSQSTHVPLTF	77	1	65
CQQHYSTPRTF	71	34	39
CQQSNEDPWTF	82	51	15
CLQHGESPFTF	48	31	73
CWQGTHFPWTF	77	40	35
CQQSKEVPWTF	102	55	4
CWQGTHFPYTF	52	71	39
CWQGTHFPRTF	43	67	54
CHQYLSSWTF	47	48	70
CQHHYGTPYTF	60	53	53
CQNGHSFPYTF	15	94	57
CQQGQSYPLTF	87	38	41
CQQWSSNPYTF	73	37	59
CWQGTHFPQTF	58	64	48
CQHHYGTPLTF	39	73	59
CQQYYSYPWTF	56	103	14
CFQGSGYPLTF	66	5	103
CFQGSHVPFTF	58	47	70
CHQYLSSYTF	35	62	78
CQQHYSTPFTF	66	61	48
CLQYASSPWTF	52	59	65
CQNDYSYPLTF	52	73	54
CQQWSSNPFTF	50	55	77
CQHFWGTPYTF	48	70	68
CQQGQSYPWTF	73	32	83
CQQYSGYPLTF	97	48	45
CQQSKEVPRTF	7	51	138
CMQHLEYPLTF	52	72	74

	Pool 1	Pool 2	Pool 3
CQQSNEDPFTF	30	120	48
CLQSDNMPYTF	60	76	69
CQHFWGTPWTF	41	69	97
CQQRSSYPLTF	69	96	42
CQQWSSYPYTF	38	45	124
CFQGSHVPRTF	30	82	97
CLQSDNLPLTF	68	79	63
CSQSTHVPFTF	60	96	67
CQQGNTLPPTF	87	36	103
CQHFWGTPLTF	122	10	100
CAQNLELPWTF	91	50	93
CQQHNEYPWTF	64	86	89
CQHHYGTPRTF	94	2	144
CLQYASYPRTF	50	78	122
CQQHNEYPLTF	84	76	93
CQQYSSYPYTF	97	101	62
CQQYNSYPFTF	131	91	46
CQQYYSYPLTF	56	93	130
CLQSDNLPYTF	97	103	80
CLQYDEFPLTF	106	82	93
CQQYYSYPYTF	128	73	80
CQQHNEYPYTF	110	87	85
CQQYSKLPYTF	108	100	74
CFQGSHVPPTF	102	103	78
CQQDYSSPYTF	136	67	80
CQQRSSYPFTF	12	143	130
CLQYASSPFTF	87	91	112
CQNGHSFPFTF	10	198	90
CSQSTHVPPTF	91	110	97
CQQSKEVPYTF	84	112	103
CQQSNEDPLTF	102	131	70
CHQRSSWTF	175	126	3
CQQSNSWPHTF	117	64	124
CLQYASSPLTF	128	103	83
CQQSNSWPTTF	122	111	85
CWQGTHFPFTF	150	58	113
CGQSYSYPYTF	106	120	103
CHQYLSSRTF	94	112	130
CQQHYSTPPTF	122	123	92
CQQGQSYPYTF	144	63	138
CQHFWGTPRTF	79	138	130
CQHHYGTPWTF	117	131	100
CWQGTHFPHTF	91	138	122
CQNDHSYPYTF	73	163	117
CSQSTHVPRTF	110	126	117
CWQGTHFPLTF	84	159	110
CQQGSSIPLTF	128	112	116
CQHFWSTPWTF	112	188	57
CQHHYGTPFTF	117	131	113
CQQYWSTPYTF	144	129	88
CQQYSSYPFTF	175	143	46
CAQNLELPYTF	112	138	117
CQNVLSTPYTF	64	143	160
CQNDHSYPLTF	136	109	124
CQQGSSIPRTF	97	191	93
CQQDYSSPLTF	144	108	130
CQHFWGTPPTF	102	89	196
CVQYAQFPYTF	131	112	144
CQHFWGTPFTF	117	96	182
CQNGHSFPRTF	4	224	167
CHQYLSSLTF	158	131	107
CQHSRELPLTF	225	87	85
CQQDYSSPWTF	136	149	117

B

	Pool 1	Pool 2	Pool 3
CQQNNEDPWTF	136	138	128
CQNVLSTPFTF	87	182	138
CQNVLSTPWTF	153	149	107
CLQYDEFPWTF	169	82	160
CLQYDEFPFTF	79	170	163
CLQYDNLRTF	144	103	167
CQHFWSTPYTF	131	182	102
CQQLVEYPFTF	163	29	223
CQQWSGYPFTF	136	129	150
CQQSKEVPPTF	175	59	188
CLQSDNMPFTF	136	151	138
CQQWSSNPWTF	122	198	111
CQQNNEDPFTF	188	198	51
CQHSRELPYTF	158	151	144
CLQSDNLPFTF	153	166	138
CQQHLHIPYTF	153	131	176
CQNGHSFPWTF	9	214	238
CHQRSSYPWTF	131	182	150
CQQGNTLPLTF	122	147	196
CLQYASYPYTF	158	159	150
CAQFYSYPLTF	214	124	130
CQNGHSFPPTF	71	239	158
CFQSNYLPYTF	73	179	223
CQNDYSYPYTF	169	96	213
CQHHYGTPPTF	188	131	160
CQQNNEDPYTF	175	166	138
CSQSTHVPPWTF	188	163	128
CQQSNEDPPTF	150	159	171
CQHFWVTPRTF	158	217	107
CQQRSSYPPTF	158	170	163
CQQLVEYPYTF	243	119	130
CAQNLELWTF	243	138	113
CKQSYNLWTF	271	217	6
CQQGSSIPFTF	169	170	155
CLQHWNYPLTF	163	179	155
CQQYWSTPLTF	203	170	124
CGQSYSYPLTF	214	170	117
CLQYDNLLTF	108	198	196
CQQNNEDPRTF	153	126	223
CLQYDNLLYTF	203	112	188
CQQYHSYPLTF	41	224	238
CQNDYSYPFTF	150	198	158
CLQYDNLLWTF	214	82	213
CLQYASYPWTF	163	198	150
CQQYYSYPRTF	112	198	203
CHQWSSYPLTF	243	239	36
CQQYWSTPWTF	169	154	196
CLQGTHQPWTF	175	143	203
CLQVTHVPYTF	175	159	188
CVQYAQFPWTF	188	163	176
CLQYDNLFTF	79	214	238
CQQLYSTPYTF	243	112	182
CQQRSSYPYTF	188	191	163
CSQSTHVPPYTF	163	182	203
CLQYASSPPTF	175	208	167
CQHSWEIPLTF	243	95	213
CLQYASSPRTF	188	182	182
CHQRSSYTF	188	28	339
CQQGNTLPFTF	188	188	182
CQQHNEYPFTF	243	264	54
CQHSWEIPYTF	225	166	182
CQQSNSWPRTF	122	191	261
CLQHGESPWTF	112	217	251

	Pool 1	Pool 2	Pool 3
CQQFTSSPYTF	203	182	196
CQQYYSYPFTF	336	101	144
CQQWSSNPPITF	203	217	171
CQQYSKLPPTF	112	271	223
CSQSTHVPTF	136	252	223
CQQYSKLPRTF	304	239	74
CQHSRELPFTF	203	239	176
CGQSYSYPFTF	304	64	261
CQHSRELPWTF	304	124	203
CQQGQSYPFTF	243	112	278
CQQDYSSPFTF	188	224	223
CFQSNYLPLTF	188	252	203
CLQYDNLLRTF	203	224	223
CQQLYSTPLTF	304	170	188
CHQRSSYPYTF	188	252	223
CHQWSSYPTF	243	170	261
CQQYSSYPTF	214	285	176
CQQYWSTPFTF	271	170	238
CVQYAQFPRTF	243	239	203
CLQYDEFPRTF	225	252	213
CQQNNEDPLTF	336	191	163
CLQYASYPFTF	336	80	278
CQQYSKLPFTF	243	191	261
CKQSYNLYTF	243	285	171
CQQLVEYPLTF	225	198	278
CKQSYNLFTF	271	120	312
CQQSNSWPWTF	271	214	223
CAQNLELPRTF	214	285	213
CAQNLELPLTF	304	208	203
CHQWSSYPYTF	225	321	171
CQQLVEYPRTF	188	321	213
CQQLYSTPWTF	225	239	261
CHQYHRSPLTF	175	239	312
CQHSRELPPTF	374	131	223
CQQYYSYPPTF	225	233	278
CKQSYNLLTF	271	252	223
CQQWSSNPRTF	442	80	238
CQHFWSTPRTF	188	239	339
CQQGSSIPYTF	203	224	339
CQQDYSSPPTF	225	264	278
CLQYDNLLFTF	336	154	278
CQNVLSTPPTF	374	264	130
CQHSWEIPWTF	225	285	261
CLQYDEFPPTF	243	217	339
CQQWSSFPFTF	136	147	518
CQQFTSSPWTF	243	321	238
CSQSTHVPPLTF	304	208	290
CQQSRKVPWTF	336	208	261
CQQYSSYPWTF	225	208	373
CLQHWNYPFTF	117	387	312
CQQYSKLPLTF	271	233	312
CQQYSGYPYTF	243	285	290
CQQSYSAPLTF	304	264	251
CLQYDEFPPYTF	163	321	339
CQQYHSYPPTF	271	321	238
CHQWSSYPPTF	304	271	261
CQQYWSTPPTF	304	271	261
CQNVLSTPRTF	175	239	425
CFQGSGYPFTF	271	233	339
CHQWSSYPWTF	336	271	238
CQHFWDTPRTF	214	321	312
CQQDYSSPRTF	225	285	339
CSQSTHVPPFTF	374	239	238

B

	Pool 1	Pool 2	Pool 3
CHQRSSYPFTF	203	427	223
CQQYWSTPRTF	243	321	290
CVQYAQFPLTF	271	271	312
CQQYNSYPRTF	374	285	196
CQNVLSTPLTF	271	252	339
CKQAYDVPYTF	304	299	261
CQQWSSFPYTF	203	154	518
CQQYSSYPRTF	243	321	312
CLQVTHVPLTF	131	472	278
CHQYHRSPPTF	225	285	373
CQQWNSYPLTF	271	472	144
CSQSTHVWTF	336	299	261
CQQGNTLRTF	374	387	150
CLQHSYLPYTF	304	357	251
CQQWSGYPYTF	243	357	312
CQQSKEVPFTF	574	321	19
CHQYHRSPYTF	243	299	373
CQNDHSYPFTF	336	357	223
CHQYHRSPWTF	304	191	425
CQQGNTLYTF	271	224	425
CLQVTHVPWTF	336	299	290
CQQWTYPLITF	336	217	373
CQQYNSYPWTF	442	299	188
CQHFWSTPFTF	271	321	339
CHQRSSFTF	188	321	425
CQQWSNYPYTF	442	154	339
CQHFWSTPLTF	336	387	213
CVQYAQFPFTF	336	387	213
CHQYHRSPFTF	214	299	425
CHQWSSYRTF	374	239	339
CQQWSSYPRTF	271	357	339
CQQWSSYPWTF	336	321	312
CALWYSNHLVF	304	357	312
CQQWSSSPFTF	243	357	373
CQQSKEVPLTF	442	357	176
CFQGSHVPTF	304	252	425
CQQYSGYPFTF	304	387	290
CLQRNAYPLTF	374	321	290
CQQRSSYPWTF	442	299	251
CQQSNSWPQYTF	271	357	373
CSQSTHVYTF	271	357	373
CQQYSSSPLTF	374	208	425
CLQHWNYPYTF	175	321	518
CQQFTSSPSTF	203	472	339
CAQNLELPFTF	442	387	188
CQQNNEDPPTF	442	264	312
CQQLYSTPRTF	442	299	278
CQQGNTLWTF	574	271	176
CQQSNSWPQTF	374	357	290
CHQWSSYPFTF	175	427	425
CLQHGESPLTF	442	357	238
CQQYHSYPRTF	374	427	238
CLQFYEFPLTF	374	299	373
CLQYASYPLTF	442	321	290
CQQSRKVPYTF	442	321	290
CQQWSGYPLTF	442	299	312
CLQGTHQPYTF	574	285	196
CQQSNSWPTLTF	304	387	373
CQQYSGYPWTF	336	357	373
CQQHYSTPTF	374	321	373
CQQDYSSYTF	243	321	518
CQQYNNYPLTF	574	271	238
CQQFTSSPFTF	574	321	203

	Pool 1	Pool 2	Pool 3
CQQYSSYRTF	163	427	518
CHQYLSLFTF	442	299	373
CQQWSSYPITF	243	357	518
CQHSRELPRTF	442	472	213
CGQSYSYPPTF	225	387	518
CAQNLELPPTF	574	321	238
CQQWSSSPLTF	374	387	373
CQHSWEIPPTF	574	252	312
CHQRSSYPLTF	374	427	339
CLQRNAYPYTF	243	472	425
CLQGTHQPPTF	442	387	312
CQQWSNYPFTF	225	233	684
CQHSWEIPFTF	442	427	290
CQHFWNTPYTF	442	472	251
CFQGSHVPHTF	442	299	425
CLQYDELYTF	243	561	373
CQQSNSWPF	336	472	373
CQQSNSWPQLTF	336	472	373
CQQYHSYPYTF	442	427	312
CHQYLSSFTF	574	357	251
CSQSTHVLTF	574	271	339
CQQGQSYPRTF	442	321	425
CQQYSSYLTF	442	387	373
CHQRSSYPCTF	304	561	339
CFQGSGYPYTF	374	151	684
CVQGTHFPYTF	374	321	518
CQQWSSYPPITF	442	357	425
CWQGTHFRTF	442	357	425
CQQWNYPYTF	271	271	684
CALWYSNHWVF	304	239	684
CQHFWSTPPTF	336	387	518
CQQRSSYPRTF	574	299	373
CQQYHSYPWTF	169	561	518
CQQWSSSPPTF	442	472	339
CQQWNYPLYTF	442	387	425
CQQSNSWLTF	271	472	518
CGQSYSYPTF	574	321	373
CAQLLELPYTF	336	561	373
CQQLYSTPFTF	374	472	425
CLQGTHQPFTF	304	285	684
CQQGNTLPPWTF	304	285	684
CLQFYEFPYTF	374	387	518
CQQWSSNPPYTF	442	427	425
CQQWSSNPPTWTF	574	357	373
CFQSNYLPFTF	574	427	312
CQQYNSYLTF	442	357	518
CHQYLSSHTF	374	427	518
CQQYNSYPLLTF	574	387	373
CQQWSSDPFTF	225	427	684
CHQYLSYTF	442	472	425
CQQHYSSPLTF	271	729	339
CQQYSTYPYTF	574	427	339
CQQYSSYYTF	442	387	518
CQQYYSYPTF	442	387	518
CLQVTHVPPTF	374	561	425
CQQHLHIPWTF	271	729	373
CVQGTHFPHTF	574	427	373
CVQGTHFPRTF	442	561	373
CKQSYNLPTF	574	285	518
CQNGHSFPF	225	729	425
CQQYSGYPLITF	304	561	518
CLQVTHVPFTF	574	472	339
CQQYSSYPLFTF	574	472	339

B

	Pool 1	Pool 2	Pool 3
CQQYYSYLTF	574	387	425
CQQWSTYPFTF	442	264	684
CWQGTHFPF	336	387	684
CQQWSSNPPMYTF	304	427	684
CHQRSSYPTF	574	472	373
CKQSYNLRTF	442	299	684
CLQHSYLPLTF	574	427	425
CAQNLELPTF	442	472	518
CQQYSKLWTF	442	472	518
CKQAYDVPLTF	374	387	684
CHQYHRSPRTF	442	321	684
CQQWSSDPYTF	574	198	684
CSQSTHIPWTF	304	729	425
CLQYDNLLPWTF	574	729	167
CQHSWEIPRTF	574	472	425
CQNDHSYPWTF	574	561	339
CVQGTHFPLTF	574	561	339
CQQYNKLPWTF	442	357	684
CQQGSSIPRITF	374	427	684
CQQYNSYPHTF	336	729	425
CQQSRKVPSTF	574	561	373
CLQYDNLLLTF	442	729	339
CQQYYTYPWTF	442	561	518
CQQYYSYRTF	374	729	425
CFQGSHVPF	374	472	684
CLQYASSLTF	374	472	684
CQQYTSYPLTF	574	285	684
CQQHLHIPFTF	304	561	684
CHQRSSYPF	442	427	684
CSQSTHVPHTF	574	299	684
CCQGSHVPLTF	574	561	425
CQQFTSSPLTF	574	561	425
CQQSYSAPFTF	574	561	425
CVQYAQFPPTF	574	561	425
CHQWSSYHTF	574	472	518
CLQYDSLWTF	574	472	518
CQQGNTLPPLTF	574	472	518
CQQWNYPLTF	574	321	684
CWQGTHFPTF	336	729	518
CHQYHRSPPITF	442	729	425
CQQRSSYPF	442	729	425
CQQWNNYPLTF	442	729	425
CSQSTHVPF	442	729	425
CQQGSSSPYTF	442	472	684
CQQWSSNPQYTF	442	472	684
CQQYYSYPF	442	472	684
CQQHYSTPF	574	729	312
CQQYNSYPF	374	561	684
CQNDYSYPPTF	374	729	518
CQQSNEDPPWTF	374	729	518
CLQYDEFPF	214	729	684
CQQWNYPFTF	225	729	684
CFQGSHVPSTF	574	561	518
CLQYDNLTWTF	574	561	518
CMQHLECPYTF	574	561	518
CQQYNSYPLYTF	574	561	518
CQQHYSTWTF	574	729	373
CQQYNSYPLAF	574	729	373
CQQYSGYPRTF	574	729	373
CLQYASSPYMYTF	442	561	684
CQQWNSNPYTF	442	561	684
CHQYLSSTF	574	729	425
CLQYDNLLPTF	574	729	425

	Pool 1	Pool 2	Pool 3
CQQWSSYPLITF	574	729	425
CALWYSNHFWVF	574	472	684
CQQHLHIPPTF	574	472	684
CSQSTHVPTWTF	374	729	684
CGQSYSYPRTF	574	561	684
CLQHGESPF	574	561	684
CLQSDNMYTF	574	561	684
CLQYDEFMYTF	574	561	684
CQQWSSNPPWTF	574	561	684
CFQGSHGPWTF	574	729	518
CHQYLSSPTF	574	729	518
CLQYASSPTF	574	729	518
CMQQLEYPYTF	574	729	518
CQQRKEVPWTF	574	729	518
CQQRSSYPPLTF	574	729	518
CQQYNSYYTF	574	729	518
CQQYSKLTWTF	574	729	518
CFQSNYLPLYTF	442	729	684
CLQYDEFPPLTF	442	729	684
CQHHYGTWTF	442	729	684
CQQYNTYPLTF	442	729	684
CCQGSHVPYTF	574	729	684
CLHYDEFPWTF	574	729	684
CLQGTYYPRTF	574	729	684
CQNDHSYPRTF	574	729	684
CQQHLHIPPLTF	574	729	684
CQQHYSTPHTF	574	729	684
CQQSIEDPYTF	574	729	684
CQQSNEDRTF	574	729	684
CQQSNSWPALTF	574	729	684
CQQSYEDPWTF	574	729	684
CQQWSSSPPITF	574	729	684
CQQYWSTALTF	574	729	684
CQQYWSTMYTF	574	729	684
